# Structural and Functional Analysis of the GRAS Gene Family in Grapevine Indicates a Role of GRAS Proteins in the Control of Development and Stress Responses

**DOI:** 10.3389/fpls.2016.00353

**Published:** 2016-03-30

**Authors:** Jérôme Grimplet, Patricia Agudelo-Romero, Rita T. Teixeira, Jose M. Martinez-Zapater, Ana M. Fortes

**Affiliations:** ^1^Instituto de Ciencias de la Vid y del Vino (Consejo Superior de Investigaciones Científicas-Universidad de La Rioja-Gobierno de La Rioja)Logroño, Spain; ^2^Faculdade de Ciências de Lisboa, BioISI, Universidade de LisboaLisboa, Portugal; ^3^Instituto de Tecnologia de Química Biológica, Biotecnologia de Células VegetaisOeiras, Portugal

**Keywords:** abiotic stress, biotic stress, fruit ripening, grapevine, GRAS gene family, transcription factor

## Abstract

GRAS transcription factors are involved in many processes of plant growth and development (e.g., axillary shoot meristem formation, root radial patterning, nodule morphogenesis, arbuscular development) as well as in plant disease resistance and abiotic stress responses. However, little information is available concerning this gene family in grapevine (*Vitis vinifera* L.), an economically important woody crop. We performed a model curation of GRAS genes identified in the latest genome annotation leading to the identification of 52 genes. Gene models were improved and three new genes were identified that could be grapevine- or woody-plant specific. Phylogenetic analysis showed that GRAS genes could be classified into 13 groups that mapped on the 19 *V. vinifera* chromosomes. Five new subfamilies, previously not characterized in other species, were identified. Multiple sequence alignment showed typical GRAS domain in the proteins and new motifs were also described. As observed in other species, both segmental and tandem duplications contributed significantly to the expansion and evolution of the GRAS gene family in grapevine. Expression patterns across a variety of tissues and upon abiotic and biotic conditions revealed possible divergent functions of GRAS genes in grapevine development and stress responses. By comparing the information available for tomato and grapevine GRAS genes, we identified candidate genes that might constitute conserved transcriptional regulators of both climacteric and non-climacteric fruit ripening. Altogether this study provides valuable information and robust candidate genes for future functional analysis aiming at improving the quality of fleshy fruits.

## Introduction

Transcription factors play an important role in the regulation of plant development and disease response. Among them, the plant gene family of GRAS transcription factors was defined based on nuclear localization, DNA binding and transcriptional activation features (Silverstone et al., 1998; Itoh et al., 2002; Morohashi et al., 2003). In addition, *in vivo* association of specific GRAS proteins with promoter regions of several putative GRAS target genes was confirmed by chromatin immunoprecipitation (Zentella et al., 2007). The name GRAS derives from its first three identified members, namely, gibberellic acid insensitive (GAI), repressor of GA1 (RGA), and scarecrow (SCR; Pysh et al., 1999; Bolle, 2004). Moreover, the Arabidopsis GRAS Protein SCL14 was shown to be essential for the activation of stress-inducible promoters (Fode et al., 2008).

Genome-wide analysis performed in nearly 30 plant species from more than 20 genera revealed that this gene family is widely distributed in the plant kingdom (Tian et al., 2004), reviewed by Hirsch and Oldroyd (2009) and it is likely to have emerged first in bacteria (Zhang et al., 2012). GRAS proteins are typically 400–700 amino acids in length and exhibit considerable sequence homology among each other in their C-terminus, where five conserved motifs, namely LHR I, VHIID, LHR II, PFYRE, and SAW are located (Pysh et al., 1999; Tian et al., 2004). The VHIID domain of a GRAS protein from *Brassica napus* interacts with a histone deacetylase, supporting the notion that GRAS proteins regulate gene expression at the level of transcription (Gao et al., 2004).

The amino acid sequences of GRAS proteins are highly variable at the N-terminus, which may be responsible for the specificity of their regulatory functions (Tian et al., 2004). For example, a subgroup of GRAS proteins, which function in several plant species as repressors of gibberellin signaling, share in their N-terminal region the amino acid sequence DELLA and are thus referred as DELLA proteins (Silverstone et al., 1998).

The GRAS protein family groups into eight well-known subfamilies: DELLA, HAM, LISCL, PAT1, LAS, SCR, SHR, and SCL3. However, in between 8 and 13 distinct clades can be discriminated in different studies (Huang et al., 2015; Bolle, 2016). Several GRAS genes from plant species such as Arabidopsis, rice, and barley have been functionally characterized, including *CIGR* (PAT subfamily), *GAI, RGL, RGA*, and *SLN1* (DELLA subfamily), *MOC1* (LAS subfamily) as well as other genes from SCR, SHR, LISCL, SCL, and HAM subfamilies (Fu et al., 2002; Stuurman et al., 2002; Day et al., 2004), reviewed by Bolle (2016). They have been involved in many processes of plant growth and development such as gibberellins signal transduction (Peng et al., 1997; Ikeda et al., 2001), axillary meristem initiation (Greb et al., 2003; Li et al., 2003), shoot meristem maintenance (Stuurman et al., 2002), radial organization of the root (Helariutta et al., 2000), phytochrome A signal transduction (Bolle et al., 2000), and male gametogenesis (Morohashi et al., 2003). *GRAS* genes have also been connected with plant disease resistance and abiotic stress response (Mayrose et al., 2006; Ma et al., 2010; Cui, 2012). Furthermore, in the model legume species *Medicago truncatula* and *Lotus japonicus* two GRAS proteins were shown to be required for nodule morphogenesis (Kalo et al., 2005; Heckmann et al., 2006). Recently, the GRAS transcription factor RAM1 and the novel GRAS protein RAD1 were reported to be involved in arbuscule development (Xue et al., 2015). The formation of multicomponent GRAS transcription factor complexes with other proteins was suggested to be a prerequisite for elicitation of nodulation or mycorrhization (Oldroyd, 2013). Genes coding for GRAS transcription factors were also identified as targets of miRNAs during tomato fruit development and ripening (Moxon et al., 2008; Karlova et al., 2013).

So far, various *in silico* genome analyses have predicted the existence of 33, 57 and 48 *GRAS* genes in the whole genome of Arabidopsis, rice and Chinese cabbage, respectively (Tian et al., 2004; Song et al., 2014). As more species have their complete reference genome sequenced, additional *GRAS* genes can be identified as it is the case of *Vitis vinifera*.

Due to its economic relevance, much research in grapevine genomics has been carried out during the last decade. Among these studies, the release of the whole grapevine genome sequence in 2007 represented a breakthrough to promote its molecular genetics analysis (Jaillon et al., 2007). Based on the published sequence data, comprehensive analysis of a given gene family can be performed to uncover its molecular functions, evolution, and gene expression profiles. These analyses can contribute to the understanding of how genes in gene families control traits at a genome-wide level.

Previous comparative analysis with Chinese cabbage genome predicted 43 GRAS transcription factors in *V. vinifera* (Song et al., 2014). In this work, we update this number to 52, a very similar number of GRAS genes to the 53 recently reported in tomato (Huang et al., 2015). Furthermore, we provide a detailed analysis of the GRAS transcription factors relationships among several plant species through comparative genomics together with the identification, structural analysis, and mapping of the GRAS transcription factors onto the grapevine chromosomes. Finally, expression analyses based on microarray and RNAseq data suggest that GRAS proteins play an important role in grape ripening and in response to abiotic and biotic stresses.

## Materials and methods

### Identification of *GRAS* genes

Genes previously identified as encoding GRAS proteins in (Grimplet et al.) were blasted (blastp and tblastn) against the grapevine genome 12x.2 (https://urgi.versailles.inra.fr/Species/Vitis/Data-Sequences/Genome-sequences), the non-redundant list of genes in (Grimplet et al., 2012) and the COST annotation gene set available at the ORCAE website (http://bioinformatics.psb.ugent.be/orcae/). Results from different analysis were manually cross-checked to identify new potential loci corresponding to *GRAS* genes in the grapevine genome. The UGene software (Okonechnikov et al., 2012) was used to design the gene models on the grapevine genome and test their structure.

### Gene structure analysis

The potential coding DNA sequences (CDS) were blasted (blastx) against the NCBI public database to compare the structures with other known *GRAS* genes in other species and the NCBI Refseq predictions of the grapevine genes. When discrepancies were observed, gene models were corrected using the UGene software. Loci bearing genes that were not functional were eliminated from the list. A GFF file with the *GRAS* genes was designed, uploaded into the IGV software and the RNAseq data available (shoot tips, leaves, flower inflorescences and seed tissues) in the laboratory were used to double-check the exon structure of the genes. Final models were uploaded in the *V. vinifera* ORCAE database (Sterck et al., 2012; Grimplet et al., 2014).

### Sequence alignment and phylogenetic analysis

Sequence information on previously reported GRAS proteins of *Arabidopsis thaliana* was retrieved from the Arabidopsis Information Resource (https://www.arabidopsis.org/browse/genefamily/GRAS.jsp). Evolutionary analyses were conducted in MEGA6 (Tamura et al., 2013). Multiple sequence alignment was inferred using MUSCLE (Edgar, 2004). The evolutionary history was inferred by using the Maximum Likelihood method based on the JTT matrix-based model (Jones et al., 1992). The bootstrap consensus tree inferred from 100 replicates was taken to represent the evolutionary history of the taxa analyzed (Felsenstein, 1985). Branches corresponding to partitions reproduced in less than 30% of bootstrap replicates were collapsed. Initial trees for the heuristic search were obtained automatically by applying Neighbor-Join and BioNJ algorithms to a matrix of pairwise distances estimated using a JTT model, and then selecting the topology with superior log likelihood value. The coding data was translated assuming a Standard genetic code table. All positions with less than 95% site coverage were eliminated. The genes were named according to Grimplet et al. (2014) based on the distance homology with Arabidopsis genes.

The alignment file between Arabidopsis and grapevine sequences was uploaded to the Jalview and UGene software for manual adjustment of the alignment and manual motif editing. Motifs identified in Tian et al. (2004) were flagged and labeled for the grapevine genes; additional motifs of high homology were also identified (at least 50% homology within the members of the subfamily on at least 10 amino acids) among grapevine sequences.

### Expression analysis

Expression data were retrieved from three different microarray platforms (Affymetrix Genchip (16k probesets) GrapeGen (21k probesets), Vitis Nimblegen array (29k probesets), and from our in-house RNAseq projects. Data normalization was performed on all the array of each platform (RMA normalization). After retrieving the values for the probesets corresponding to each gene, the values for the 3 or 4 replicates of the same condition were averaged to obtain a total of 256 conditions (organ, cultivar, treatment, platform). Based on expression data of the grapevine gene expression atlas (Fasoli et al., 2012), a plant ontology ID was attributed to each gene if expression intensity in a tissue was above a defined threshold of absolute log2 value of 8 or absolute value of 256. The same data were used for the co-expression analysis with the whole set of genes available on the Nimblegen platform. Hierarchical clustering with Pearson correlation as metric and average linkage cluster method was performed. Genes considered as having the same profile should present a distance threshold between each other lower than of 0.2.

For further evaluation of gene expression samples corresponding to several stages of grapevine development and ripening and several abiotic and biotic stress conditions were used (Cramer et al., 2007; Deluc et al., 2007; Espinoza et al., 2007; Grimplet et al., 2007; Pilati et al., 2007; Tattersall et al., 2007; Fung et al., 2008; Lund et al., 2008; Albertazzi et al., 2009; Pontin et al., 2010; Sreekantan et al., 2010; Carvalho et al., 2011; Fortes et al., 2011; Tillett et al., 2011; Vega et al., 2011; Diaz-Riquelme et al., 2012; Fasoli et al., 2012; Lijavetzky et al., 2012; Carbonell-Bejerano et al., 2013; Agudelo-Romero et al., 2015). Heat maps were performed with the ComplexHeatmap R package (https://github.com/jokergoo/ComplexHeatmap).

### Comparison to other plant species

We performed a sequence comparison using the GRAS genes from 16 plant species (*A. thaliana, Brassica rapa, Carica papaya, Eucalyptus grandis, Citrus sinensis, Malus domestica, Prunus persica, Fragaria vesca, Glycine max, M. truncatula, Cucumis melo, Populus trichocarpa, Solanum lycopersicum, Zea mays, Sorghum bicolor, Oryza sativa*) retrieved at http://planttfdb.cbi.pku.edu.cn. We identified orthologous genes in genomes from the sixteen species following what was performed in Jaillon et al. (2007). Each pair of predicted gene sets was aligned with the BLASTp algorithm, and alignments with an e-value lower than 1e^−20^ and sequence homology higher than 40% were retained. If a comparison is above that value, the two genes were considered homologs. Two genes, A from Vitis genome GV and B from genome GX, were considered orthologs one-to-one if B was the best match for gene A in GX and A was the best match for B in GV. A phylogenetic tree was constructed with the GRAS genes from these species with the same parameters as before.

## Results

### Identification and structural annotation of the GRAS genes

Genes that were previously identified as GRAS in the grapevine genome (Grimplet et al., 2012) were used to performed sequence comparison analyses, either against the most up to date gene predictions from CRIBI V1 and V2, the NCBI refseq (on the 12Xv1 of the genome assembly) and the VCOST (on the 12Xv2 of the genome assembly) as well as directly against the reference genome sequence to check whether any potential gene could had been missed by these predictions. In this way, we identified 80 genome regions that shared homology with at least one of the genes.

Gene models were curated using the data collected from gene structure comparisons using different databases as well as the available RNAseq data from our laboratory (Royo et al., 2016) to validate actually expressed exons. This data also allowed evaluating the expression of newly detected genes, not represented in microarray data, by redoing the bioinformatics analysis of original RNAseq data with an updated GFF file. A total of 52 *GRAS* genes with a functional structure were identified in the grapevine genome (Table 1). Data relative to the detection of *GRAS* genes in previous genome annotations or gene-sets are summarized in Supplementary Table 1. Three additional genes were detected compared to the automatic annotation CRIBI V1, one was not seen in the V1, but was known in the annotation from the 8x genome (Table 1). The structure of 14 genes CRIBI annotated genes was curated in our work.

**Table 1:** **Genome localization of the 52 grapevine *GRAS* genes**.

**Locus ID**	**Short name**	**Strand**	**Position v2**	**Locus ID**	**Short name**	**Strand**	**Position v2**
Vitvi12g00665	*PAT1*	−	8738265–8739902	Vitvi11g00409	*RGA3*	−	3959545–3961143
Vitvi19g00619	*PAT2*	−	7772106–7773743	Vitvi01g01509	*GRASV1b*	+	20426662–20428254
Vitvi10g00271	*PAT3*	−	2802206–2803843	Vitvi17g01040	*GRASV1d*	−	12688373–12689932
Vitvi19g00392	*PAT4*	−	5276148–5277899	Vitvi19g01706	*GRASV1a*	+	23595896–23597488
**Vitvi16g01086**	*PAT6*	+	19383904–19385748	Vitvi14g01510	*GRASV1c*	−	25316395–25317516, 25317604–25318488
Vitvi04g01696	*PAT7*	−	23747087–23748793	Vitvi07g00627	*SCR3*	+	6996793–6998256
Vitvi18g01210	*PAT8*	+	13411198–13412895	Vitvi03g01226	*SCR2*	+	19152243–19153571
Vitvi07g01612	*SHR5*	+	21912240–21913607	**Vitvi08g00007**	*SCR1*	−	115793–116261, 116647–117596
Vitvi12g00571	*SHR4*	+	7509331–7510668	Vitvi06g01133	*SCL3a*	+	15915179–15916597
Vitvi09g01487	*SHR3*	−	910682–912319	**Vitvi02g00974**	*SCL3b*	+	13518884–13519177
Vitvi05g01554	*SHR2*	+	23894334–23895644	Vitvi13g01556	*SCL3c*	−	24957576–24959012
Vitvi07g02073	*SHR1*	−	21633666–21635150	*Vitvi14g01348*	*SCL3d*	+	*23412635–23413888*
Vitvi06g00491	*LISCL1*	+	5938487–5940601	**Vitvi18g01322**	*GRAS8b*	+	14926630–14927997
Vitvi06g00490	*LISCL4*	+	5930838–5932814	**Vitvi02g00370**	*GRAS8a*	+	3323726–3325756
Vitvi06g00492	*LISCL2*	+	5942791–5944119	**Vitvi04g01281**	*LAS2*	+	18563606–18565456
**Vitvi06g01569**	*LISCL12*	+	5918887–5921169	Vitvi19g00932	*LAS1*	+	10747971–10749212
Vitvi06g00489	*LISCL3*	+	5925910–5928204	Vitvi07g00418	*GRASV2a*	−	4408615–4410429
Vitvi08g01214	*LISCL5*	+	14792851–14795082	**Vitvi05g00110**	*GRASV2b*	−	1038770–1039128, 1039236–1039324, 1039452–1040602
Vitvi13g00312	*LISCL6*	+	3256665–3258887	*Vitvi08g00751*	*GRASV3a*	−	*9219561–9220028, 9220132–9221151*
Vitvi13g00314	*LISCL8*	+	3283478–3285724	*Vitvi08g00746*	*GRASV3b*	−	*9152326–9153933*
Vitvi13g00311	*LISCL9*	+	3251727–3254009	**Vitvi08g01969**	*GRASV3c*	+	9227520–9229388
Vitvi13g01865	*LISCL10*	+	3279518–3281677	**Vitvi04g01622**	*SCL26a*	+	22393173–22394627
**Vitvi13g01864**	*LISCL11*	+	3274050–3274663, 3274680–3276222	Vitvi18g00300	*SCL26b*	−	3254592–3256064
**Vitvi13g00313**	*LISCL7*	+	3270544–3270684, 3270692–3271508, 3271694–3271929, 3271938–3272162	Vitvi04g01247	*HAM3*	−	18244582–18246198
Vitvi01g00446	*RGA5*	−	4895406–4897178	**Vitvi02g00536**	*HAM1*	+	5144861–5147299
**Vitvi14g00841**	*RGA4*	+	14807005–14808846	Vitvi15g00680	*HAM2*	−	14397074–14399326

*Bold IDs correspond to genes which CDS structure was curated regarding v1 annotation. Italics indicate the gene is new when compared to v1 annotation. Genes VviSCL3d, VviGRASV3a, and VviGRASV3b correspond to newly detected genes compared to V1. VviSCL3d was already known in the 8x genome. The 14 CRIBI annotated genes with curated structure in this work are in bold*.

Exon/ intron structure is highly conserved amongst *GRAS* genes in grapevine and most of them presented only one exon which is a common feature of this gene family observed in many plant species (Song et al., 2014; Huang et al., 2015; Lu et al., 2015). Only six genes contained introns (Table 1). Five of them contained two exons while *VviLISCL7* contained four. No subfamily showed a specific intron/exon structure (Supplementary Table 1) while the size of *GRAS* genes varied greatly, ranging from 294 nucleotides (*VviSCL3b*) to 2349 nucleotides (*VviSCR1*). Forty-one genes (79%) had a length longer than 1400 bp.

### Phylogenetic analysis, nomenclature, and motif analysis

**Figure 1:**
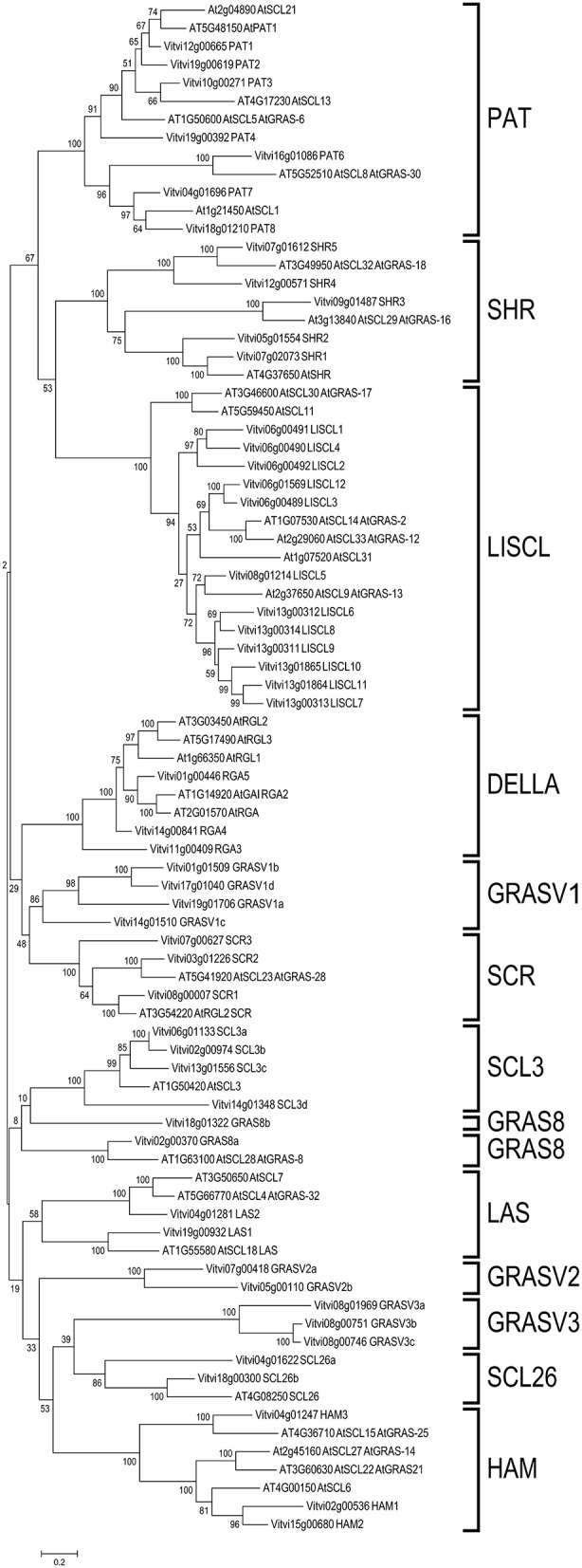
**Molecular phylogenetic analysis of grapevine and Arabidopsis *GRAS* genes**. Thirteen sub families were identified in grapevine: the known DELLA, HAM, LISCL, PAT, LS, SCR, SHR, and SCL3 and five new subfamilies GRAS8, GRASV1, GRASV2, GRASV3, and SCL26.

For gene nomenclature, a phylogenetic tree of the GRAS protein coding genes in *V. vinifera* and Arabidopsis was constructed (Figure 1) as recommended by the Super-Nomenclature Committee for Grape Gene Annotation (sNCGGa; Grimplet et al., 2014). This analysis identified the eight subfamilies previously described in other plant species: DELLA, HAM, LISCL, PAT, LS, SCR, SHR, and SCL3. Furthermore, five additional groups were detected that could not been assigned to any of those subfamilies (Figure 1). Interestingly, 13 groups were also recently found in tomato (Huang et al., 2015). For individual gene nomenclature, we attributed gene symbols/names using preferentially those previously used when they fit the recommendations of the sNCGGa. If a gene was not described before and had an Arabidopsis ortholog, the corresponding Arabidopsis gene name was used. In addition, to distinguish different subfamily members, names were composed by the subfamily symbol followed by a number or a letter (when the subfamily symbol ended with a number). Among the new detected subfamilies, two showed an Arabidopsis homolog that had not been previously described in a subfamily. These were labeled SCL26 and GRAS8. The 3 remaining new subfamilies were labeled GRASV1, GRASV2 and GRASV3.

**Figure 2:**
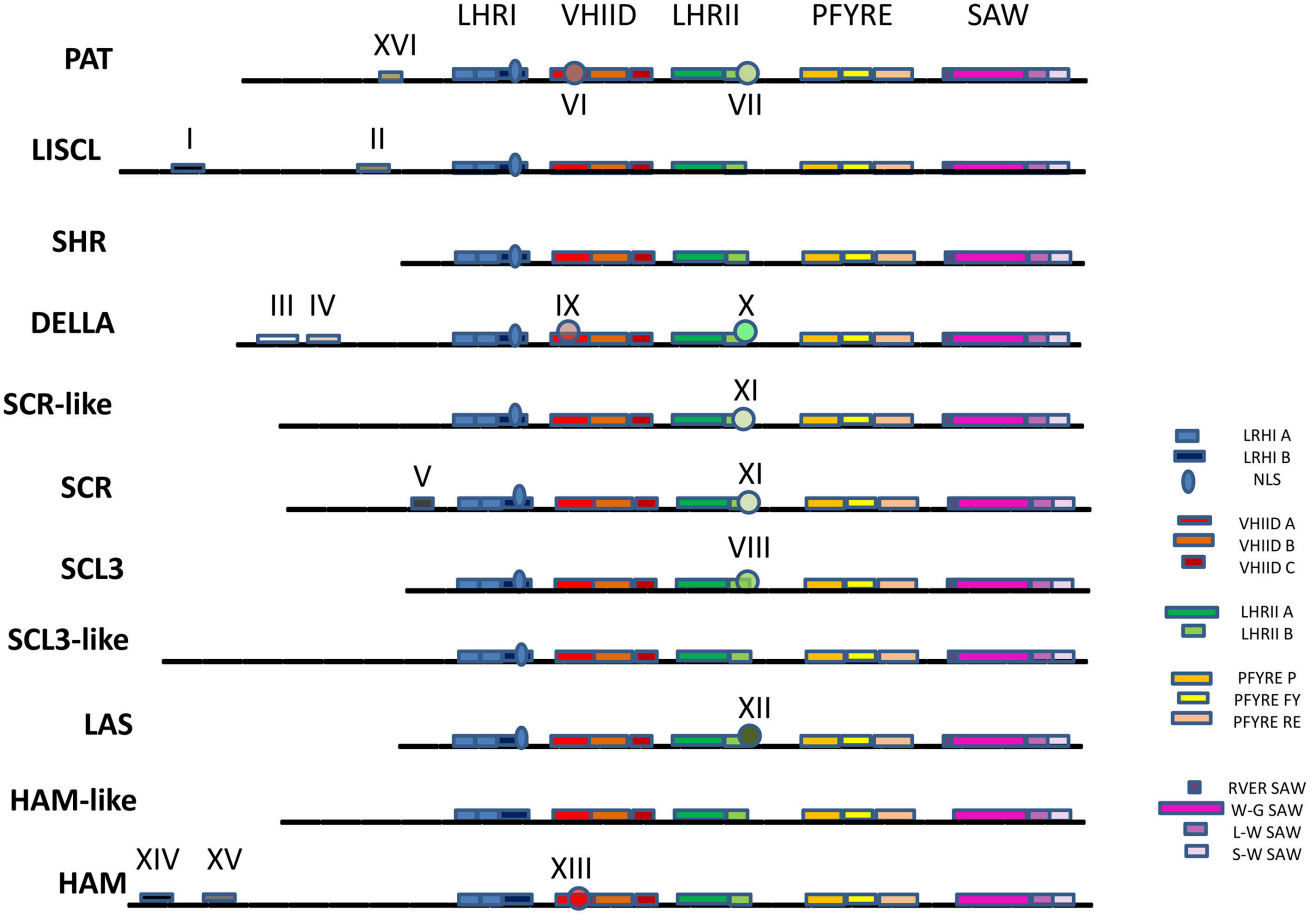
**Structure and subfamily-specific motifs of GRAS proteins**. The five conserved motifs LHR I, VHIID, LHR II, PFYRE, and SAW are displayed. Subfamily-specific motifs are labeled with roman numerals. GRASV1 (SCR-like) does not present domain V. GRAS8 (SCL3-like) does not present domain VIII. GRASV2, V3, and SCL26 (HAM-like) do not present domains XIII, XIV, and XV. HAM and HAM-like sequences do not show NLS. Complete details on gene structure are shown in Supplementary Image 1.

Five characteristic conserved motifs were identified in the C-terminus of the GRAS proteins, namely LHRI, VHIID, LHRII, PFYRE, and SAW (summarized by subfamilies in Figure 2 and detailed in Supplementary Image 1). The LHRI motif presented two units (A and B). Leucine repeats found in Unit A were found to be conserved in all GRAS proteins (Figure 2 and Supplementary Image 1) as previously reported (Tian et al., 2004). Unit B contained a putative nuclear localization signal (NLS). The canonical NLS was present in the cluster of DELLA proteins in the phylogenetic tree (Figure 1) though it appeared degenerated in *VviRGA3* (Supplementary Image 1).

The VHIID motif contained three units (A, B, and C). GRAS proteins could be divided into several distinct groups based on conservation of Unit A. Groups such as PAT, DELLA, and HAM presented high conservation of amino acids (VI, IX, and XIII respectively, Figure 2). Unit B was extremely conserved and the C unit had a conserved pattern of LRITG (Pysh et al., 1999; Tian et al., 2004). The L was substituted by I or V and in the case of DELLA proteins by F unit.

The LHRII motif embraced units A and B. In Unit A, three regularly spaced leucine heptad repeats (LX6LX6L) could be found followed by several irregularly spaced leucine repeats. In Unit B, many GRAS proteins had a conserved LXXLL pattern (DELLA, SCL3, and LS groups) as previously described (Tian et al., 2004; Figure 2 and subgroups X, VIII, and XII). The PAT1 and SCR groups presented different conserved patterns (VII and XI).

The PFYRE motif could be divided into three units: P, FY, and RE. On the other hand, the SAW motif was composed of two units, RVER and W-W-W (Figure 2). RVER could be noticed in many but not all GRAS proteins. Members in the HAM subfamily lacked the RVER domain in their C-termini as well as some members of the SHR group (Figure 2 and Supplementary Image 1). The W-W-W unit included three subunits: W-G, L-W, and S-W (Figure 2).

In the N-terminus several units were found, in accordance with previous reports (Tian et al., 2004). Units I and II of the LISCL group, units III and IV of DELLA proteins, and unit V of SCR group (Figure 2). Only one sequence in Arabidopsis (AtRGL2) and its ortholog in *V. vinifera* presented domain V in the SCR group. The TVHYNP domain is characteristic of DELLA proteins (unit IV). In two *V. vinifera* sequences (VviLISCL2 and VviLISCL7) the domains I and/or II of LISCL proteins were missing due to the fact that the N-terminus is too short (Supplementary Image 1). The N region was much conserved in LISCL. The N- terminus of SHR proteins was also very short. Furthermore, in HAM subfamily we identified two new motifs named XIV and XV and in PAT subfamily a new motif named XVI (Figure 2). The consensus sequences for the new motifs are for XIV: TSVLDTRRSPSPPTSTSTSTL+SS++GGG; and for XV: ++EQS+L+WI+GDV+DPS+G; XVI: RELE+ALLGPDDDD).

Besides these eight known groups, five new additional groups were identified. A new *V. vinifera* group (formed by four proteins- VviGRAS V1a-Vd) showed similarity with SCR proteins but lacked the SCR motif (Figures 1, 2). This new subfamily was not present in Arabidopsis and was named GRASV1, with V for *Vitis*. However, this subfamily is apparently only absent in Arabidopsis and Brassica as observed in a comprehensive phylogenetic analysis that includes grapevine and fifteen other plant species (Supplementary Image 2).

A subgroup of proteins with much similarity to the SCL group did not present VIII domain including *AtGRAS8* and its ortholog in *V. vinifera* (*VviGRAS8*). Roman numeric nomenclature for subfamilies as used in Lu et al. (2015) was considered confounding since it was also used to label the motifs, so this subfamily was renamed as VviGRAS8, following the name of the Arabidopsis gene.

Based on the original phylogenetic analysis (Figure 1) we detected a third subfamily apparently related to the Arabidopsis gene *SCL26* but the broad species analysis (Supplementary Image 2) revealed that this subfamily should be split in 3 distinct subfamilies since only two genes were grouped with *SCL26* in the species analysis. All these proteins were also phylogenetically related to the HAM subfamily but lacking the XIII domain, a reason why they were not included in the HAM group. Furthermore, we identified GRASV2 and GRASV3 subfamilies within the HAM-like group. Both gene subfamilies had representative genes in other species (Supplementary Image 2).

From the alignment of predicted GRAS domain sequences we identified members containing partial GRAS domains with missing motifs (Supplementary Image 1). The gene *VviSCL3b* seemed severely truncated, it presented a premature stop codon lacking the motifs PFYRE and SAW). Interestingly, this gene whose predicted protein has 98 aminoacids is homologous to *SlGRAS35* which only contains 85 aminoacids Huang et al., 2015.

**Figure 3:**
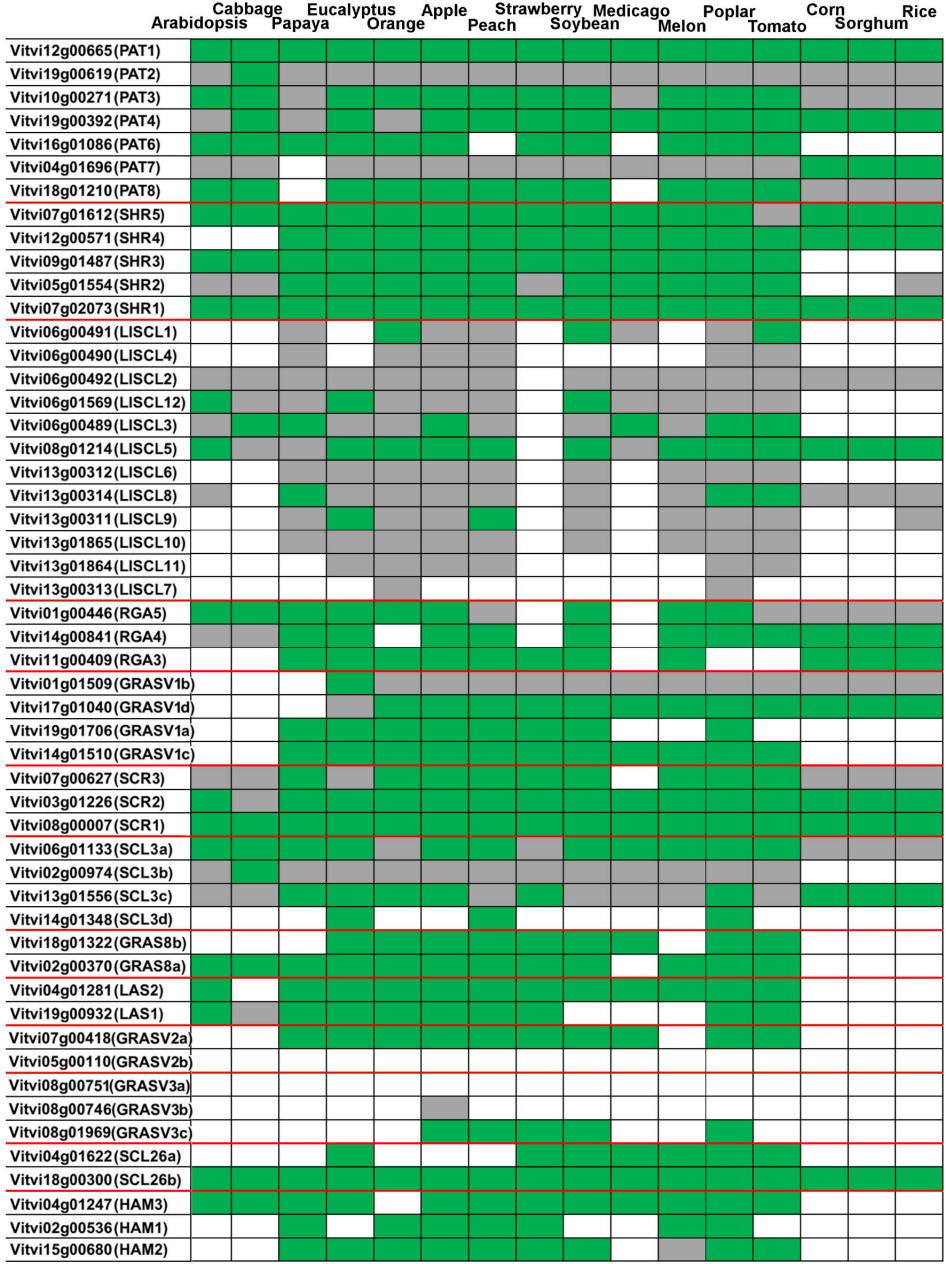
**Grapevine GRAS genes orthology against plant species with sequenced genome**. Green: a one-to-one ortholog in the species (ortholog one-to-one = best match in the species that has the grapevine deduced protein as the best match in grapevine.). Gray: the grapevine deduced protein has homology in the species genome but no one-to-one ortholog was detected (the best match do not have the grapevine deduced protein as best match). White: no match in the species.

As mentioned previously we analyzed the orthologous relationships of GRAS genes in *V. vinifera* and other species (Figure 3 and Supplementary Image 2). The orthologous relationships were classified into three categories: (i) genes present in grapevine and absent in a given species; (ii) grapevine genes showing a one-to-one relationship with one gene from a given species; (iii) grapevine genes having homologs in a given species, but without no clear putative ortholog (Figure 3). When grapevine genes were compared only to *Arabidopsis*, 18 genes showed a one-to-one ortholog relationship with an Arabidopsis gene, a value slightly higher to the 15 obtained in the comparative analysis performed between *Prunus mume* and *Arabidopsis* (Lu et al., 2015). These genes likely correspond to well-conserved functions between both species. Eleven grapevine genes had homologs in Arabidopsis but no one-to-one relationship could be found. On the other hand, 23 genes do not have homologs in Arabidopsis.

A phylogenetic tree considering several mono and dicotyledonous species together with a sequence comparison were performed to identify genes with widely conserved functions among species (Figure 3). Genes that might represent evolutionary conserved functions were *VviPAT1, VviSHR1, VviSCR1*, and *VviSCL26g* since orthologs were found in all the species analyzed (Figure 3).

GRAS gene family has considerably evolved since the divergence of monocot and eudicot plants as determined by the orthologous relationship of GRAS genes in several species. The phylogenetic analysis of LISCL, HAM, PAT, and SCL groups revealed independent clusters with many members from only monocotyledonous species (Supplementary Image 2). On the other hand, *E. grandis and P. trichocarpa* putative specific subgroups were also noticed. GRAS family expanded significantly in these fast-growing woody tree species. According to Liu and Widmer (2014) there are 106 and 94 GRAS genes in Populus and Eucalyptus, respectively. In *V. vinifera* no species-specific subgroup was found.

Regarding the new *V. vinifera* subfamilies, the results indicated that group comprising VviGRASV1a-Vd, existed before the divergence of dicots and monocots and were lost in Arabidopsis and *B. rapa* (Figure 3 and Supplementary Image 2). However, *VviGRASV1c* and *VviGRASV1d* did not appear in monocots.

The genes *VviGRASV2a*- and *VviGRASV3c* also presented orthologs in some species but not in Arabidopsis and *B. rapa*. The gene *VviGRASV2a* is homologous to two genes from tomato (Supplementary Image 2); therefore they may eventually play similar functional roles in fleshy fruits such as grapevine and tomato. Orthologs of *VviGRASV2a* can be found in many other species whereas for *VviGRASV2b* no ortholog was detected (Figure 3).

Regarding the GRAS8 subfamily, gene *VviGRAS8a* was included in a large cluster with *AtSCL28* and homologous genes in tomato and rice. It has orthologs in several species including tomato but not in rice. *VviGRAS8b* has homologs in several mono and dicotyledonous species but not in Arabidopsis and *B. rapa*. Orthologs were not found in Arabidopsis and monocots.

*VviSCL26b* clustered with *AtSCL26* and several other species whereas *VviSCL26a* did not have homologs/orthologs in Arabidopsis. As expected, since they were never described before in other species, the genes from the new families' shared little homology with genes from Arabidopsis.

### Chomosomal location of the GRAS genes

**Figure 4:**
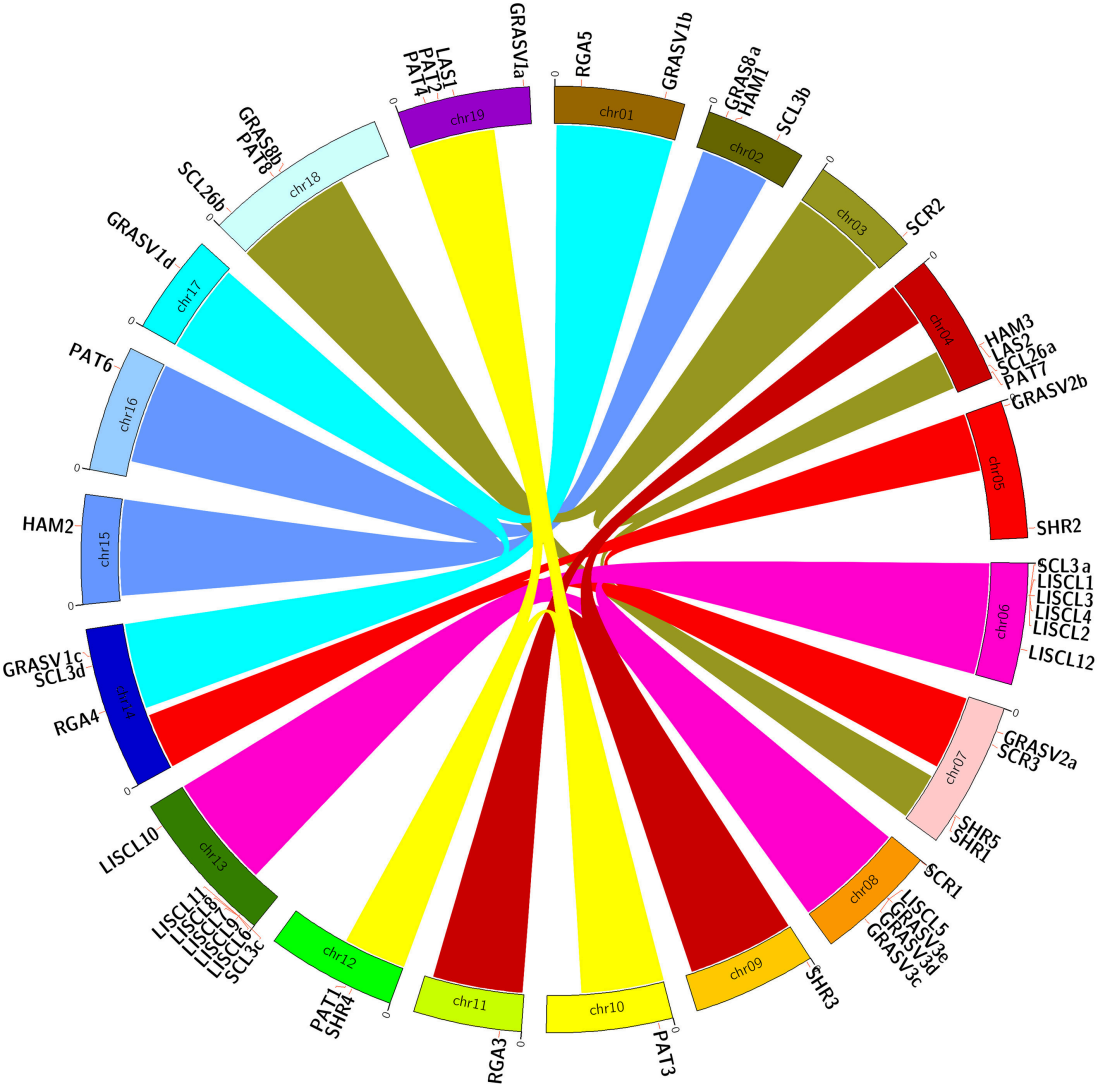
**Chromosomal location of grapevine GRAS genes**. Links with the same colors in different chromosomes show paralogous regions as previously defined (Jaillon et al., 2007).

GRAS genes were distributed unevenly among the nineteen chromosomes of the grapevine genome though they were mapped to all the chromosomes (Figure 4). The highest number of GRAS genes was found on chr 6 and 13, with 6 and 7 genes respectively. The high number of GRAS sequences in these two chromosomes is mainly due to the presence of repeats of genes belonging to the same group (LISCL). On the other hand, chr 3, 9, 10, 11, 15, 16, and 17 only bore one gene. GRAS genes belonging to the same group were located in chromosomal regions that may represent paralogous segments resulting from ancestral polyploidization events (Jaillon et al., 2007; Velasco et al., 2007). LISCL genes were located in chr 6, 8, and 13 (although most of the LISCL in chr 13 were located just beside the presumed paralogous segment) and PAT genes located in chr 10, 12, and 19.

Concerning LISCL genes, the tandem repetition of almost identical coding sequences (e.g., *VviLISCL7* and *VviLISCL11*) suggests that these duplication events in the grapevine genome are quite recent (Licausi et al., 2010). There is also tandem repetition of genes belonging to different groups such as *VviLISCL5* and *VviGRASV3c-e* as well as *VviSCL3a*, and *VviLISCL1-4*). Interestingly, clusters in chr 6 and 13 presented similar sequence string within 4 LISCL genes followed by one SCL3.

Tandem repeats mainly in the LISCL group were also observed in *P. mume* (Lu et al., 2015).

Interestingly, the new *V. vinifera* group comprising *VviGRAS Va-Vd* was distributed in four different chromosomes (1, 14, 17, and 19). Three of them were in paralogous regions in chr 1, 14, and 17.

Therefore, segmental duplication and tandem duplications contributed significantly to the expansion and evolution of the GRAS gene family.

### Expression analysis of grapevine GRAS genes

**Figure 5:**
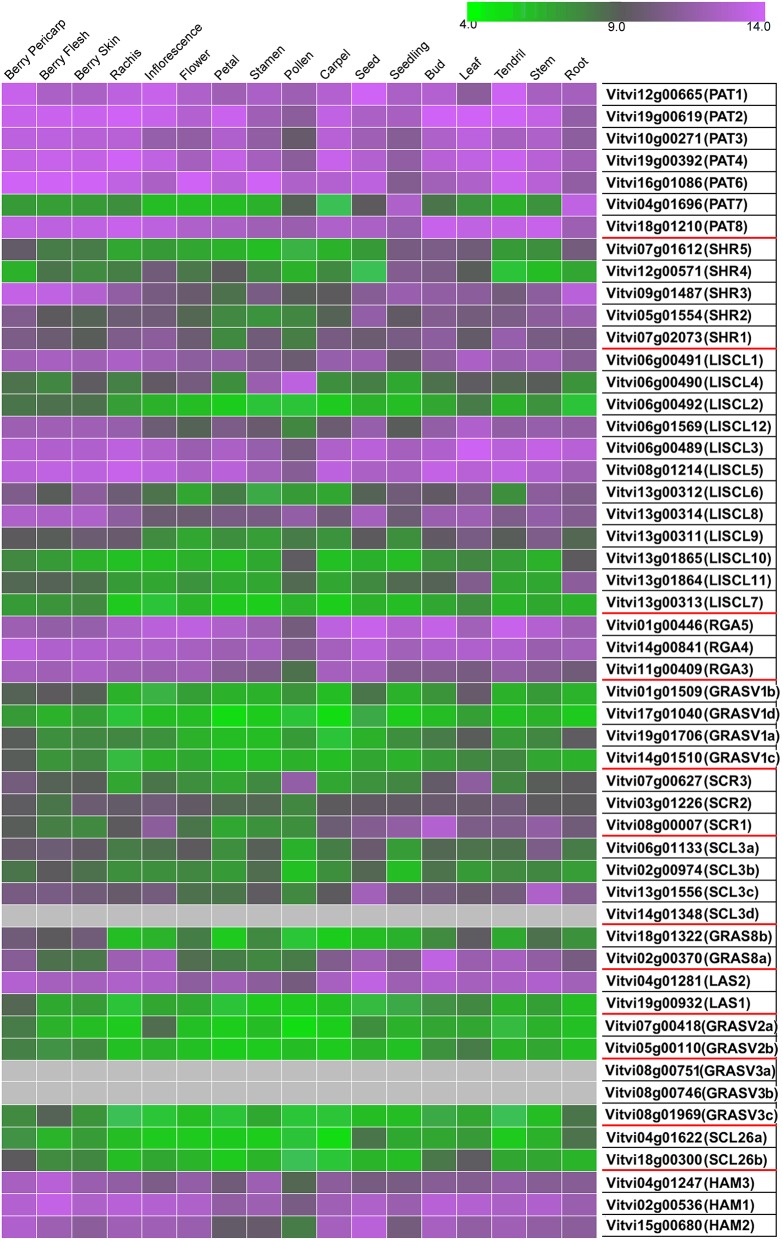
**Expression of GRAS genes in grapevine tissues**. Gradient color is expressed in RMA-normalized intensity value on the Nimblegen microarray. The value for each tissue corresponds to the condition where the highest expression was reported.

Three distinct approaches were performed to characterized GRAS genes expression in grapevine. First, we constructed an atlas of expression of the GRAS genes based on the absolute value of gene expression in public data. The results of this study are presented in Figure 5 that displays the data extracted from the published grapevine gene expression atlas (Fasoli et al., 2012). When a gene was clearly expressed in a given tissue a Plant Ontology (PO) was attributed to the gene and reported in the ORCAE database.

Second, we performed a co-expression analysis based on the same original data using the relative values of expression of all the genes, centered on the average expression. The objective here was to determine expression patterns and to identify genes that were following the same pattern of expression as the GRAS genes and that could be under the same regulatory elements, or under the regulation of the GRAS gene itself. The results are presented in Table 2 and Supplementary Table 2. Nine genes showed a correlation with other genes with a Pearson Correlation Coefficient (PCC) threshold of 0.2. Finding the optimal PCC threshold to retrieve functionally related genes was affected by the method of gene expression database construction and the target gene function (Obayashi and Kinoshita, 2009), but the PCC that was chosen was very stringent.

**Table 2:** **Co-expression analysis of GRAS genes**.

**Unique_ID/nimblegen probeset**	**Functional_annotation**	**Functional categories**
VIT_02s0025g04000	***VviGRAS8a***	**GRAS family transcription factor**
VIT_14s0068g02000	Ribonucleotide reductase R2	Nucleotide metabolism. Purine metabolism
VIT_11s0016g03750	Myb-related protein 3R-1 (Plant c-MYB-like protein 1)	Cellular process. Cell growth and death
VIT_18s0001g07550	Kinesin family member 4/7/21/27	Microtubule-driven movement
VIT_13s0064g00560	DNA topoisomerase, ATP-hydrolyzing	Nucleic acid metabolism. DNA metabolism
VIT_18s0122g00550	Cyclin-dependent kinase B2;1	Cell growth and death; Regulation of cell cycle
VIT_14s0108g00710	Chromosome condensation protein	DNA metabolism. DNA replication
VIT_11s0016g02970	MAP kinase kinase 6	Signaling pathway. Protein kinase. MAPK cascade
VIT_13s0067g03250	CENP-E like kinetochore protein	Cellular process. Cell growth and death
VIT_13s0067g01420	Cyclin 1b (CYC1b)	Cell growth and death; Regulation of cell cycle
VIT_06s0004g05870	Tubulin beta-3 chain	Microtubule organization and biogenesis
VIT_18s0001g02060	Cyclin A1	Cell growth and death; Regulation of cell cycle
VIT_07s0005g01030	Cellulose synthase CSLD5	Cell wall biosynthesis. Cellulose biosynthesis
VIT_01s0010g02430	Mitotic spindle checkpoint protein (MAD2)	MAPK cascade; Regulation of cell cycle
VIT_12s0057g00500	Thymidine kinase	Nucleotide metabolism. Pyrimidine metabolism
VIT_13s0019g02710	Rho guanyl-nucleotide exchange factor ROPGEF5	Signaling pathway. G-protein signaling pathway
VIT_04s0008g01080	Calmodulin-binding region IQD6	Calcium sensors and Signaling
VIT_14s0068g00270	Hydroxyproline-rich glycoprotein	Cell wall organization and biogenesis
VIT_10s0003g05680	CHUP1 (chloroplast unusual positioning 1)	Cytoskeleton. Actin organization and biogenesis
VIT_04s0023g01660	***VviLAS2***	**GRAS family transcription factor**
VIT_12s0059g00230	Epoxide hydrolase 2	Epoxide hydrolase family; Biotic stress response
VIT_12s0059g00220	Epoxide hydrolase	Epoxide hydrolase family; Biotic stress response
VIT_08s0007g02240	Calcium/proton exchanger CAX3	Electrochemical Potential-driven Transporters. Porters. Ca2+:Cation Antiporter
VIT_05s0020g03380	WNK1 (with no lysine (K) 1)	Signaling pathway. Circadian clock Signaling
VIT_14s0108g01420	DEFENSE NO death 1	Biotic stress response. Plant-pathogen interaction
VIT_12s0035g00970	Evolutionarily conserved C-terminal region 11 ECT11	RNA processing. mRNA processing. mRNA splicing
VIT_02s0025g04120	Calmodulin binding protein	Signaling pathway. Calcium sensors and Signaling
VIT_04s0023g01170	Unknown protein	Unknown
VIT_03s0180g00140	Acetyl xylan esterase AxeA	Unknown
VIT_10s0003g02780	Unknown protein	Unknown
VIT_05s0020g00870	UbiE/COQ5 methyltransferase	Biosynthesis of derivatives of dehydroquinic acid, shikimic acid and chorismic acid
VIT_01s0244g00140	Aspartate kinase	Amino acid. Glycine, serine, and threonine metabolism
VIT_07s0005g03700	***VviSCR3***	**GRAS family transcription factor**
VIT_15s0046g00930	Zinc finger (C3HC4-type ring finger)	Transcription factor. Zinc finger C3HC4 family transcription
VIT_07s0129g00030	***VViSHR1***	**GRAS family transcription factor**
VIT_08s0007g04820	Pectate lyase	Cell wall catabolism. Pectin catabolism
VIT_07s0129g01070	Leucine-rich repeat protein kinase	Signaling. Signaling pathway. Protein kinase
VIT_02s0025g02700	Glutaredoxin family protein	Response to stimulus. Stress response. Abiotic stress
VIT_18s0001g09920	Cyclin delta-3 (CYCD3_1)	Cytokinin-mediated Signaling pathway
VIT_12s0059g01900	Unknown protein	Unknown
VIT_01s0026g01420	Wall-associated kinase 4	Signaling. Signaling pathway. Protein kinase
VIT_01s0137g00720	Lipase GDSL	Unclear
VIT_07s0005g00740	Endo-1,4-beta-glucanase	Cell wall catabolism. Cellulose catabolism
VIT_09s0002g00450	Subtilase	Subtilase-mediated proteolysis
VIT_05s0077g02270	Unknown protein	Unknown
VIT_18s0001g07340	Aspartic proteinase nepenthesin-1 precursor	Proteolysis. Peptidase-mediated proteolysis
VIT_03s0038g02180	Glycosyl hydrolase family 10 protein	Cell wall catabolism. Xylan catabolism
VIT_14s0030g01870	NIMA protein kinase	Signaling. Signaling pathway. Protein kinase
VIT_01s0010g01660	Receptor protein kinase	Signaling. Signaling pathway. Protein kinase
VIT_08s0056g00050	***VViSCR1***	**GRAS family transcription factor**
VIT_18s0001g10380	Heat shock transcription factor B4	HSP-mediated protein folding; Temperature stress response
VIT_09s0002g01540	Unknown protein	Unknown
VIT_04s0044g01100	Invertase/pectin methylesterase inhibitor	Cell wall organization and biogenesis
VIT_11s0016g04630	***VviRGA3***	**GRAS family transcription factor**
VIT_08s0007g02760	IAA-amino acid hydrolase 1 (ILR1)	Auxin activation by conjugation hydrolysis
VIT_13s0019g01780	***VviLISCL11***	**GRAS family transcription factor**
VIT_10s0003g02350	SRG1 (senescence-related gene 1) oxidoreductase	Unclear
VIT_13s0019g01810	***VviLISCL8***	**GRAS family transcription factor**
VIT_07s0005g05640	Unknown protein	Unknown
VIT_18s0001g03310	***VviSCL26b***	**GRAS family transcription factor**
VIT_13s0067g01190	Cellulase	Cell wall catabolism. Cellulose catabolism
VIT_03s0088g00890	Pathogenesis related protein 1 precursor [*Vitis vinifera*]	Jasmonate-mediated Signaling pathway; Biotic stress response. Plant-pathogen interaction
VIT_05s0094g01310	Polygalacturonase GH28	Cell wall modification. Pectin modification
VIT_10s0092g00070	Taxane 13-alpha-hydroxylase	Diterpenoid biosynthesis
VIT_08s0105g00170	Dof zinc finger protein DOF3.5	C2C2-DOF family transcription factor
VIT_05s0124g00210	Peptidase S26A, signal peptidase I	Proteolysis. Peptidase-mediated proteolysis
VIT_05s0062g00690	Heat shock protein 81-2 (HSP81-2)	HSP-mediated protein folding; Biotic stress response. Plant-pathogen interaction
VIT_15s0021g01590	RKL1 (Receptor-like kinase 1)	Signaling. Signaling pathway. Protein kinase
VIT_03s0091g00890	Endoxylanase	Cell wall organization and biogenesis
VIT_12s0055g00980	Peroxidase precursor	Phenylalanine biosynthesis; Abiotic stress response. Oxidative stress response

*The list of co-expressed genes is complete except for VviGRAS8a and VviSCL26b. Further details are presented in Supplementary Table 2. The list of co-expressed genes are highlighted in bold*.

**Figure 6:**
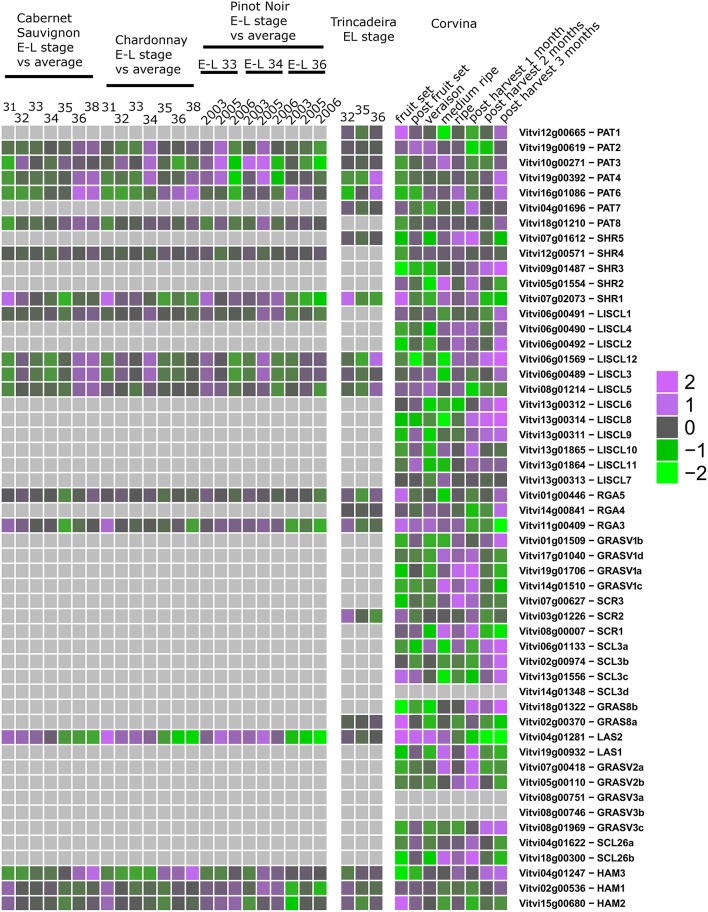
**Expression of GRAS genes during grape development and ripening**. Left: experiments performed with GeneChip microarrays. Middle: experiment performed with Grapegen microarrays. Right: experiment performed with Nimblegen microarrays.

**Figure 7:**
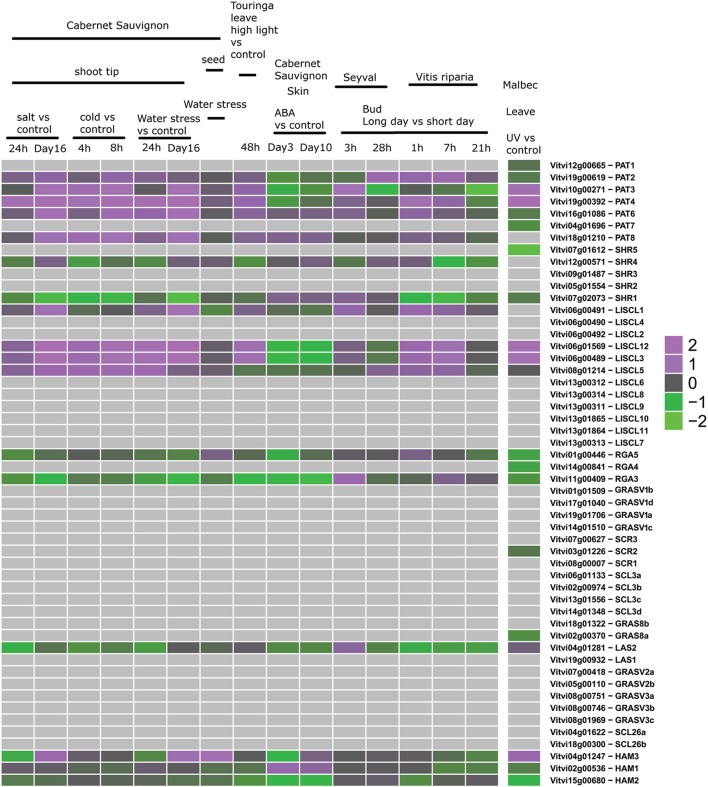
**Expression of GRAS genes upon abiotic stress**. Left: experiments performed with GeneChip microarrays. Right: experiment performed with Grapegen microarrays.

**Figure 8:**
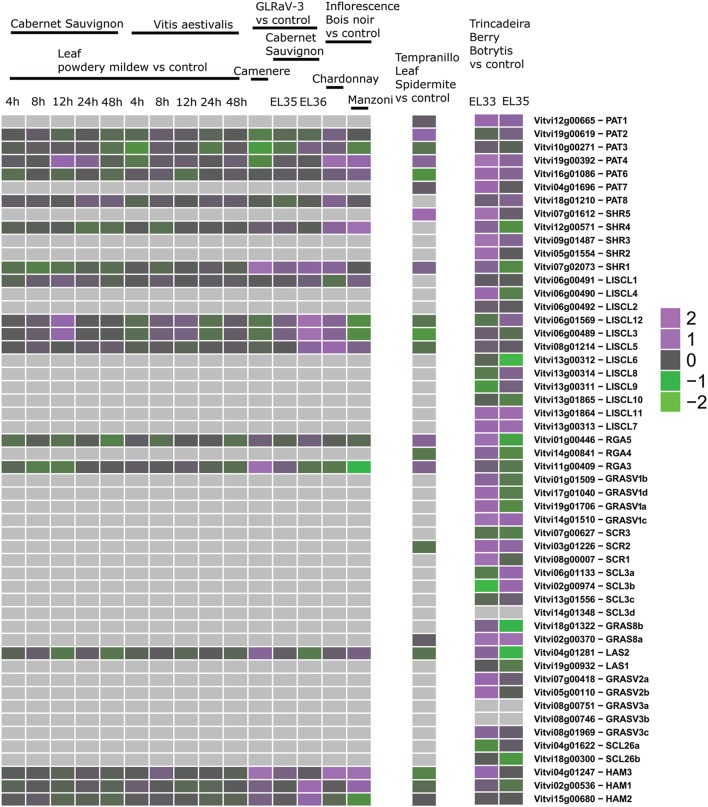
**Expression of GRAS genes upon biotic stress**. Left: experiments performed with GeneChip microarrays. Middle: experiment performed with Grapegen microarrays. Right: experiment performed with Nimblegen microarrays.

Third, we mined public expression data to identify the behavior of *GRAS* genes during berry ripening (Figure 6) and upon abiotic and biotic stresses (Figures 7, 8) not only in *V. vinifera* but also in other *Vitis* species (Supplementary Table 3). Figures 6–8 presented the expression values among the experiments where difference in expression of GRAS genes was detected.

Out of the 52 genes analyzed, six were not detected in any analyzed tissue. The rest of the genes mostly showed a general pattern; they were either highly expressed or lightly expressed in all tissues considered. Nevertheless, about one third of the genes showed some tissue-specific expression. Pollen stands out as a different tissue in terms of GRAS genes expression. Differential expression of some GRAS genes among different tissues was previously shown for tomato and Populus (Liu and Widmer, 2014; Huang et al., 2015). Furthermore, differential expression was clearly noticed during grape ripening and stress response.

#### PAT subfamily

Expression studies of *VviPAT* genes showed that most of them were expressed in all the tissues, including berry, seed, inflorescence, flower and rachis, among others (Figure 5). *VViPAT6* seemed to be more abundant in reproductive organs (flower, stamen, tendril and berry). *VViPAT7* was expressed only in seedling and root. *VviPAT* genes generally seemed to respond to abiotic stress specifically *VviPAT3, VviPAT4*, and *VviPAT6* were up-regulated after prolonged exposure (Figure 8). *VViPAT3* and *VviPAT4* also seemed to respond to photoperiod and showed a stronger expression under UV light. *VviPAT4* was up-regulated in grapevine response to *Botrytis cinerea*, leaf response to powdery mildew and inflorescence response to *Bois Noir* suggesting that it could be an important regulator of biotic stress responses (Figure 8). *VViPAT3, VviPAT4*, and *VViPAT6* were expressed along grape ripening (Figure 6) although differences could be noticed among cultivars and ripening stages (Supplementary Table 3). Data on the evolution during ripening confirmed that their expression seems dependent of environmental factors since expression did not seem reproducible over the years in Pinot Noir. However, their expression clearly increased in ripe fruit suggesting that these genes might be related to ripening control.

#### SHR subfamily

Concerning SHR subfamily, *VviSHR1, VviSHR2*, and *VviSHR3* tended to be expressed in all tissues excepted in some floral organs and pollen (Figure 5). *VviSHR4* and *VviSHR5* seemed to be expressed only in specific vegetative tissues. *VviSHR4* showed expression in seedling and *VviSHR2* in stem and root. *VviSHR3* showed the strongest expression in seedling, root and berry. This gene together with *VviSHR5*, an ortholog of AtSCL32, was up-regulated in berries upon *Botrytis cinerea* infection (Figure 8). *VviSHR4* responded positively to *Bois Noir* attack. *VviSHR1* was expressed in several reproductive and vegetative tissues ranging from reproductive tissues (inflorescence and carpel) to root, among others. *VviSHR1* presented co-expression with a cluster of 15 genes that included genes involved in cell wall catabolism, defense, and signaling pathways (Table 2). During ripening, its expression appeared higher during the earlier stages and seemed to be lower at *véraison*. In post-harvest berries this gene was also down-regulated.

#### LISCL subfamily

Members of the LISCL subfamily showed distinctive expression patterns. *VviLISCL3, VviLISCL5, VviLISCL8*, and *VviLISCL12* were expressed in all tissues but pollen, while *VviLISCL2, VviLISCL7, VviLISCL10* were expressed in almost none tissue (Figure 5). Among them, *VviLISCL2* expression seemed restricted to older tissues since it was only detected in post-harvest fruit, senescent leave and woody stem. The other genes presented a tissues-specific expression. Expression of *VviLISCL4* was predominant in male reproductive tissues (stamen and pollen).

*VviLISCL3* and *VviLISCL12* originated from a duplication event and have high sequence similarity, which resulted in not having a specific probeset for each of them in the GeneChips array. However, their expression seemed to be affected by ripening with the lowest expression around or after *véraison* and the highest expression in ripe or overripe stages (Figure 6). They showed high expression under prolonged abiotic stress and upon virus infection, but distinction between both genes could not be made. Nevertheless, UV light surely affected their expression positively. *VviLISCL1* was also over-expressed after 16 days under water deficit and salt stress (Figure 7).

Interestingly, *VviLISCL7*, whose expression was not detected in most tissues, showed slight over-expression upon Botrytis infection (Figure 8). Although *VviLISCL7* presented a short N-terminal lacking domain I, it might be still functional because it looked expressed in some particular conditions, with motifs II, LHRI, VHIID, LHRII, PFYRE, SAW, and RVER (unit B of LHRII was also missing). *VviLISCL2* also presented a short N-terminal lacking domain I and II; therefore some motifs may not be essential for functionality. *VviLISCL11* showed coexpression with a senescence- related gene (Table 2) and was over expressed in post-harvest berries.

#### DELLA subfamily

Genes *VviRGA3, VviRGA4*, and *VviRGA5* were expressed in all tissues (Figure 5). *VviRGA3* and *VviRGA5* were up-regulated in the earliest stages of fruit development, at fruit set and might be involved in the transition from inflorescence to flower. *VviRGA3* was also down-regulated under abiotic stresses namely salt, water stress, ABA exposure and high light (Figure 7). *VviRGA3* co-expressed with an auxin biosynthesis-related coding for gene IAA-amino acid hydrolase (Table 2), and might be a key regulator of this enzyme. Moreover, their highest expression was detected in plant tissues commonly responsible for auxin production such as seed and flower. *VviRGA5* was up-regulated in berries infected with Botrytis at green stage but its expression severely dropped at *véraison* so it might participate only in the early response (Figure 8).

#### SCR subfamily

The gene *VviSCR3* showed peaks of expression in pollen, ripe berries and senescing leaves (Figure 5) and co-expressed with a Zinc finger transcription factor (C3HC4 family). Interestingly, *VviSCR2*, an ortholog of *AtSCL23*, was down- regulated during ripening in both Trincadeira and Corvina (Figure 6). *VviSCR1*, an ortholog of *AtRGL2*, was expressed only in some vegetative tissues (seedling, bud and stem) but was slightly up-regulated in green berries upon Botrytis infection and showed a dramatic shift of expression between *véraison* and medium ripe stage in Corvina. This gene co-expressed with a heat shock transcription factor and an invertase/pectin methylesterase inhibitor (Table 2).

#### SCL3 subfamily

Three SCL3 genes (*VviSCL3a, VviSCL3b, VviSCL3c*) showed similar expression patterns (Figure 5). They were predominantly expressed in the stem, seed and berry flesh. Particularly, *VviSCL3c* might be involved in seed development. The three genes were also up-regulated in late post-harvest withering stages (Figure 6). Furthermore, *VviSCL3b* was up-regulated upon Botrytis infection in Trincadeira grapes at *véraison* stage (Figure 8). No expression was found for *VviSCL3d* which only had orthologs in papaya and peach. This gene could be a pseudogene that lost its function during the evolution of the gene subfamily.

#### GRAS8 subfamily

In this subfamily, *VviGRAS8a*, an ortholog of *AtSCL28/GRAS8*, exhibited detectable expression in several tissues ranging from inflorescence to tendril and stem (Figure 5). *VviGRAS8a* was down-regulated during grape ripening in Corvina, while no differences were observed in Trincadeira (Figure 6). In a general manner, *VviGRAS8a* was more abundant in young tissues (leaf, stem, tendril, rachis, bud) with the only exception of seed. This gene was co-expressed with a large set of genes (79 genes); most of them annotated as genes involved in cell cycle, microtubule organization, nucleotide metabolism or signaling (Table 2 and Supplementary Table 2). This suggests that it might play a role in cell growth and differentiation. It was also over-expressed at ripening and slightly up-regulated upon Botrytis infection in Trincadeira grapes. On the contrary, *VviGRAS8b* was expressed in older tissues (increased expression during post-harvest stages of ripening, leaf, stem, winter bud). As for *VviGRAS8a*, the exception was in the seed where no difference between young and old tissues was noticed.

#### LAS subfamily

Genes *VviLAS1* and *VviLAS2* presented quite a different expression profile with *VviLAS1* not being expressed in most tissues (Figure 5). *VviLAS2* appeared to be more abundant at the beginning of fruit development, with consistency among varieties. *VviLAS1* was over expressed in mature berries but not in over-ripe berries (Figure 6). *VviLAS2* expression also decreased upon Botrytis infection (Figure 8) and co-expressed with 11 genes, some of them possibly involved in biotic stress response (Table 2 and Supplementary Table 2).

#### GRASV1, GRASV2, GRASV3, and SCL26 subfamilies

Expression of genes belonging to these new subfamilies was low. For some of them, their possible expression could not be confirmed (*VviGRASV1d, VviGRASV3a, VviGRASV3b*, although for the latter two we only had RNAseqdata for expression validation). The *VviGRASV1* genes shared a similar expression profile during Corvina ripening, peaking at the medium-ripe or ripe stage and showing expression in the first post-harvest stage (Figure 6). *VviGRASV2* genes also showed this profile. Interestingly, *VviGRASV1* and *VviGRASV2* genes might also play a role during Botrytis attack (Figure 8).

*VviGRASV3c* was mostly expressed in post-harvest berries. In addition, these 2 subfamilies did not show expression in other tissues, with the exception for *VviGRASV3c* in root and *VviGRASV2a* in young inflorescence.

The SCL26 genes showed a reduced expression level in various tissues. Most notably *VviSCL26b* seemed more abundant in berries at ripe stage (Figure 6). *VviSCL26b* co-expressed with genes involved in the pathogen response and in cell wall metabolism but the function of many of the co-expressed genes was unknown (Table 2, Supplementary Table 2). The expression profile of these genes was intriguing since little consistency was observed among replicates of the same condition. This inconsistency might be caused by a response to unidentified factors during sampling, which appears in experiments performed by independent laboratories.

#### HAM subfamily

This subfamily is present in all tissues with notable lower values in pollen (Figure 5). *VviHAM3* was up-regulated during ripening, upon *Bois Noir* attacks, and in response to drought in the seed and shoot tip (Figures 6–8). *VviHAM1* and *VviHAM2* were down-regulated in all the cultivars during ripening; they might play a role in early stages of fruit development.

## Discussion

The availability of sequenced genomes, expression data and associated bioinformatics tools enable the study of the genomic information to predict the putative function of a gene family in developmental processes and in stress response. In general, transcription regulators belonging to the same taxonomic group exhibit common evolutionary origins and specific conserved motifs related to molecular functions, making their genome-wide analysis an effective and practical method to predict unknown protein functions.

We have performed an exhaustive analysis of *GRAS* genes on the 12x grapevine genome sequence based on the isolation of the complete set of genes identified in PN40024. Chromosome localization, gene structure analyses, phylogenetic analyses with other genome sequenced species and expression analysis allowed to propose an extended characterization of the *GRAS* gene family in grapevine and to draw hypotheses on the function of newly described genes.

### Expansion of GRAS family in grapevine

The grapevine *GRAS* gene family was greatly expanded by segment/chromosomal duplications as it occurred in other species belonging to different taxonomic groups (Liu and Widmer, 2014; Huang et al., 2015; Lu et al., 2015). Duplicated genes might show functional redundancy and their identification may contribute to decipher gene functions, the evolutionary consequences of gene duplication and their contribution to evolutionary change. Duplicated genes face one of these fates: nonfunctionalization, neofunctionalization (evolving novel functions), or subfunctionalization (partition of gene functions; Prince and Pickett, 2002). The process of non-functionalization can occur when a redundant gene degenerates to a pseudogene or is lost from the genome due to the vagaries of chromosomal remodeling, locus deletion or point mutation (Prince and Pickett, 2002). Likely candidate pseudogenes are some of the outliers in our sequence alignments such as gene *VviSCL3b* which presents only 294 nucleotides and a premature stop codon and lacks motifs PFYRE and SAW. Interestingly, this gene showed an ortholog only in cabbage (Figure 3). However, this gene was found to be expressed suggesting that it could still maintain some functionality. No expression was found for *VviSCL3d* which may also be a pseudogene that lost its function during the evolution of the gene family.

We have also identified duplicated grapevine genes such as *VviLISCL7* and *VviLISCL11* whose expression analysis with specific probes might indicate they have evolved into distinct functions. Expression divergence in duplicated *GRAS* gene was previously detected in several plant species (Wu et al., 2014). Furthermore, no *GRAS* genes were coexpressed together, reflecting a wide diversity of the functions, or specialization. Unlike other species, tandem duplication events in grapevine seemed mainly restricted to the LISCL subfamily which contained tandem repeated genes with the highest homology. However, other genes from specific subfamilies were in paralogous areas of the genome resulting from polyploidization event (Jaillon et al., 2007). Amongst them, the PAT subfamily had members in chr 10, 12, and 19 (Figure 4), GRASV1 in chr 1, 14, and 17, LISCL in chr 6, 8, 10, and GRASV2 in chr 5 and 7 (only two genes). Although *V. vini*fera has a smaller size genome than *S. lycopersicum* (487 and 760 Mb, respectively), it contained a similar number of *GRAS* genes (52 and 53 genes, respectively). In addition, *P. mume* with a genome size of 280 Mb, almost half the size of the *V. vinifera* genome, contained 46 *GRAS* genes, a close number to the 52 *V. vinifera* genes (Lu et al., 2015). Therefore, the density of *GRAS* genes varies greatly among plant species (Song et al., 2014; Huang et al., 2015; Lu et al., 2015).

The exon-intron organization analysis showed that 88.46% (46 out of 52) of *VviGRAS* genes were intronless in grapevine, the highest percentage found so far, though similar to *P. mume* (82.2%) (Lu et al., 2015). Interestingly, this percentage is much smaller in Populus (54.7%) where the GRAS family greatly expanded (Liu and Widmer, 2014). Horizontal gene transfer of plant GRAS genes that originated from prokaryotic genomes has been proposed (Zhang et al., 2012). This prokaryotic origin followed by extensive duplication events in their evolutionary history might explain the abundance of intronless genes within the *GRAS* gene family. The grapevine GRAS genes also exhibited a highly variable N-terminal domain, as in other species, indicating the functional versatility of this gene family in grapevine. By contrast, highly conserved C-terminal domains (GRAS domain) were observed in all non-truncated proteins.

### GRAS family members are putatively involved in grapevine development and defense

#### Expression patterns across a variety of tissues revealed divergent functions

*GRAS* genes showed broad expression patterns across a variety of tissues, as previously observed in *Populus* and *P. mume* (Liu and Widmer, 2014; Lu et al., 2015). For example, *VviSCR1* was highly expressed in the bud whereas the other *VviSCR* genes were not detected in this tissue. In Arabidopsis, *SCR* was located downstream of *SHR*, and both genes were required for stem cell maintenance of the root meristem to ensure its indeterminate growth (Lee et al., 2008). In *V. vinifera, VviSHR3* was the gene from SCR and SHR subfamilies presenting highest expression in the root. Its tomato ortholog (*SlGRAS16*) also displayed its highest expression in the root comparing to several tissues and organs tested and was also predicted to be involved in root development (Huang et al., 2015).

*VviSCR1*, ortholog of *AtSCR*, co-expressed with an invertase/pectin methylesterase inhibitor putatively involved in cell wall organization and biogenesis. *VviSHR1* was expressed in several reproductive and vegetative tissues and was co-expressed with a cluster of genes putatively involved in cell wall biogenesis (pectate lyase, endo-1,4-beta-glucanase, glycosyl hydrolase family 10 protein) and signaling mechanisms (leucine-rich repeat protein kinase, receptor protein kinase, wall-associated kinase 4). Previous analysis of a short-root (*shr*) mutant showed that the AtSHR protein is also involved in root and shoot radial patterning (Helariutta et al., 2000). These transcription factors are likely to play a role in cell wall reorganization and signaling events during cell growth and differentiation in grapevine. *SHR* and *SCR* were referred to be expressed in leaves, in young leaf primordia, in developing leaf vascular tissue, and bundle sheet cells (reviewed by Bolle, 2016). Recently, *AtSHR, AtSCR*, and *AtSCL23* were described to control bundle sheath cell fate and function in *A. thaliana* and this developmental pathway seemed to be evolutionarily conserved (Cui et al., 2014). AtSCR was identified as primarily involved in sugar transport whereas *AtSCL23* might play a role in mineral transport. Their expression seemed regulated by SHR protein. Their orthologs in *V. vinifera* (*VviSHR1, VviSCR1*, and *VviSCR2*, respectively) might play similar cellular functions. The tomato genes *SlGRAS25* and *SlGRAS15* (respective orthologs of *VviSHR1* and *VviSCR1*) in addition to *SlGRAS39*, ortholog of another SHR gene, *VviSHR2*, showed high mRNA expression levels in root and stem (Huang et al., 2015), suggesting conserved functions with their homologous gene *AtSHR* (Cui et al., 2007), and *AtSCR* (Helariutta et al., 2000) which are involved in root and shoot radial patterning in Arabidopsis. These genes had orthologs in most species (Figure 3) indicating that their function might also be conserved in grapevine.

GRAS proteins have also been involved in axillary meristem development. Knock-out Arabidopsis plants for *AtLAS/SCL18* are unable to form lateral shoots during vegetative development (Greb et al., 2003). In tomato, mutant plants for the ortholog lateral suppressor (*LeLs*) were blocked in the initiation of axillary meristems and showed lower number of flowers per inflorescence, absence of petals, reduced fertility, and altered hormone levels (Schumacher et al., 1999). The grapevine ortholog (*VviLAS1*) was not expressed in most tissues, except for berry pericarp, mature berry and leaf; however the other member of this subfamily, *VviLAS2*, showed tissue expression that could be more in accordance to the role described for *LeLs*. The ortholog of *VviLAS2* in tomato (*GRAS17*) is also differentially expressed from mature green stage fruits to breaker stage fruits (Huang et al., 2015).

In grapevine, *VviHAM1* is strongly expressed during fruit set and in several tissues such as bud, leaf, and stem. In the petunia mutant hairy meristem (ham) shoot apical meristems fail to retain their undifferentiated character (Stuurman et al., 2002). In Arabidopsis, the GRAS proteins from the HAM branch (SCL6, 22, and 27) are also involved in leaf development (Wang et al., 2010). *VviHAM1* may be involved in the regulation of meristematic activity in growing tissues.

Many *VviPAT* genes showed expression in a wide range of tissues and might be involved in several developmental processes, through the regulation of phytochrome signaling mechanisms, as in Arabidopsis (Bolle, 2004, 2016). PAT genes *PAT1, SCL5, SCL21* are positive regulators of phytochrome-A signal transduction while *SCL13* is mainly involved in phytochrome-B signal transduction (Bolle et al., 2000; Torres-Galea et al., 2006, 2013). The grapevine PAT subfamily showed the weakest expression in the less photosynthetic tissues (pollen, roots), with the exception of *VviPAT7* that displayed an opposite expression profile. *VviPAT7* was also one of the few PAT genes with no orthology in other species, except in monocots.

DELLA genes presented a wide range of expression patterns among tissues consistent with their role as negative regulators of gibberellin signal transduction (Peng et al., 1997; Silverstone et al., 1998; Zentella et al., 2007). They interfere with a variety of growth and developmental processes such as stem elongation, flower development, and seed germination (Bolle, 2004). In addition, DELLA proteins integrate not only gibberellin -signaling pathways but also jasmonate, auxin, brassinosteroid, and ethylene pathways, constituting a main signaling hub (Wild et al., 2012; Bolle, 2016). *VviRGA5*, a one-to-one ortholog of *AtRGA/AtGAI*, was highly expressed in seed, flower and stem supporting a role in developmental processes.

The rice DLT gene modulates brassinosteroid-related gene expression (Tong et al., 2009). The homologous gene in Arabidopsis is *AtSCL28* and in *V. vinifera VviGRAS8a*. Interestingly, this gene co-expressed with a large set of genes involved in cell cycle, nucleotide metabolism or signaling. In general, the transcripts of this gene were more abundant in young tissues (leaves, stem tendril, rachis, bud) and in inflorescence which is not surprising since brassinosteroids promote growth (reviewed by Fortes et al., 2015). The tomato ortholog *SlGRAS41* was suggested to be involved in flower-fruit transition with a potential role in fruit development by modulating brassinosteroid signaling (Huang et al., 2015). A role that is likely to be played by *VviGRAS8a* in grapevine eventually through an involvement in mechanisms of cell division and differentiation.

As previously mentioned, expression of GRAS genes in pollen tissue differed from other tissues. *VviLISCL4* was almost specifically expressed in the stamen and particularly in pollen. Interestingly, a *LISCL* gene has been shown to be involved in transcriptional regulation during microsporogenesis in the lily anther (Morohashi et al., 2003). Future functional analysis of *VviLISCL4* gene during pollen development is required to confirm the importance of this GRAS gene in grapevine reproduction.

Several GRAS genes (*VviLISCL2, VviGRASV2b*) showed higher expression in senescent tissues (senescent leaves, woody stem, post-harvest berries) than in younger tissues, including ripe/mature tissues. In this way, a wheat *LISCL* gene, *TaSCL14*, was identified as promoting senescence in leaves (Chen et al., 2015). *VviGRASV2b* seemed completely grapevine-specific and its potential involvement in senescence has yet to be clarified.

#### GRAS are likely to play a role in berry development and ripening

Several grapevine *GRAS* genes showed differential expression among berry ripening stages (Fortes et al., 2011; Agudelo-Romero et al., 2013) namely *VviLISCL3/12, VviLISCL11, VviPAT3, VviPAT4, VviPAT6, VviSCR3, VviGRAS8b, VviLAS1, VviHAM3, VviSCL26b* (up-regulated), *VviHAM1, VviHAM2, VviRGA3, VviSHR1, VviLAS2* (down-regulated). Genes *VviHAM1, VviHAM2, VviRGA3, VviSHR1*, and *VviLAS2* seemed to be involved in fruit set and in the early stages of fruit development when there is intense cell division activity and sugar transport. During these stages, the levels of phytohormones such as auxins, cytokins, gibberellins, and jasmonic acid also peaked (reviewed by Fortes et al., 2015), that might be related to the up-regulation of *RGA3* since DELLA proteins integrate several phytohormone- signaling pathways (Bolle, 2016). Furthermore, *RGA3* co-expressed with a gene coding for IAA-amino acid hydrolase 1 involved in auxin metabolism (auxin activation by conjugation hydrolysis) supporting the role of *VviRGA3* in hormonal regulation.

*VviLISCL3/VviLISCL12, VviPAT4, VviPAT6*, and *VviHAM3* were up-regulated at mature stages (ripe, harvest, and post-harvest) whereas *VviSCR3* was up-regulated in medium ripe and ripe berries and co-expressed with a gene coding for a Zinc finger protein (C3HC4-type ring finger). These transcription factors have been previously described as being modulated during grape ripening (Fortes et al., 2011). *VviGRAS8b* was over-expressed at post-harvest stages and *VviLAS1* and *VviSCL26b* at medium ripe, ripe and initial post-harvest stage. The gene *VviSCL26b* co-expressed with genes involved in pathogen response (pathogenesis related protein 1 precursor, heat shock protein 81-2, peroxidase precursor) and cell wall metabolism (endoxylanase, polygalacturonase GH28, cellulase). This could be associated to the activation of genes that are related to biotic stress response as well as cell wall rearrangements taking place during grape ripening (Fortes et al., 2011). *VviLISCL11* was over expressed in post-harvest berries and might be linked to the regulation of cell wall degradation processes. In agreement with this hypothesis, it was co-expressed with a senescence related gene.

**Figure 9:**
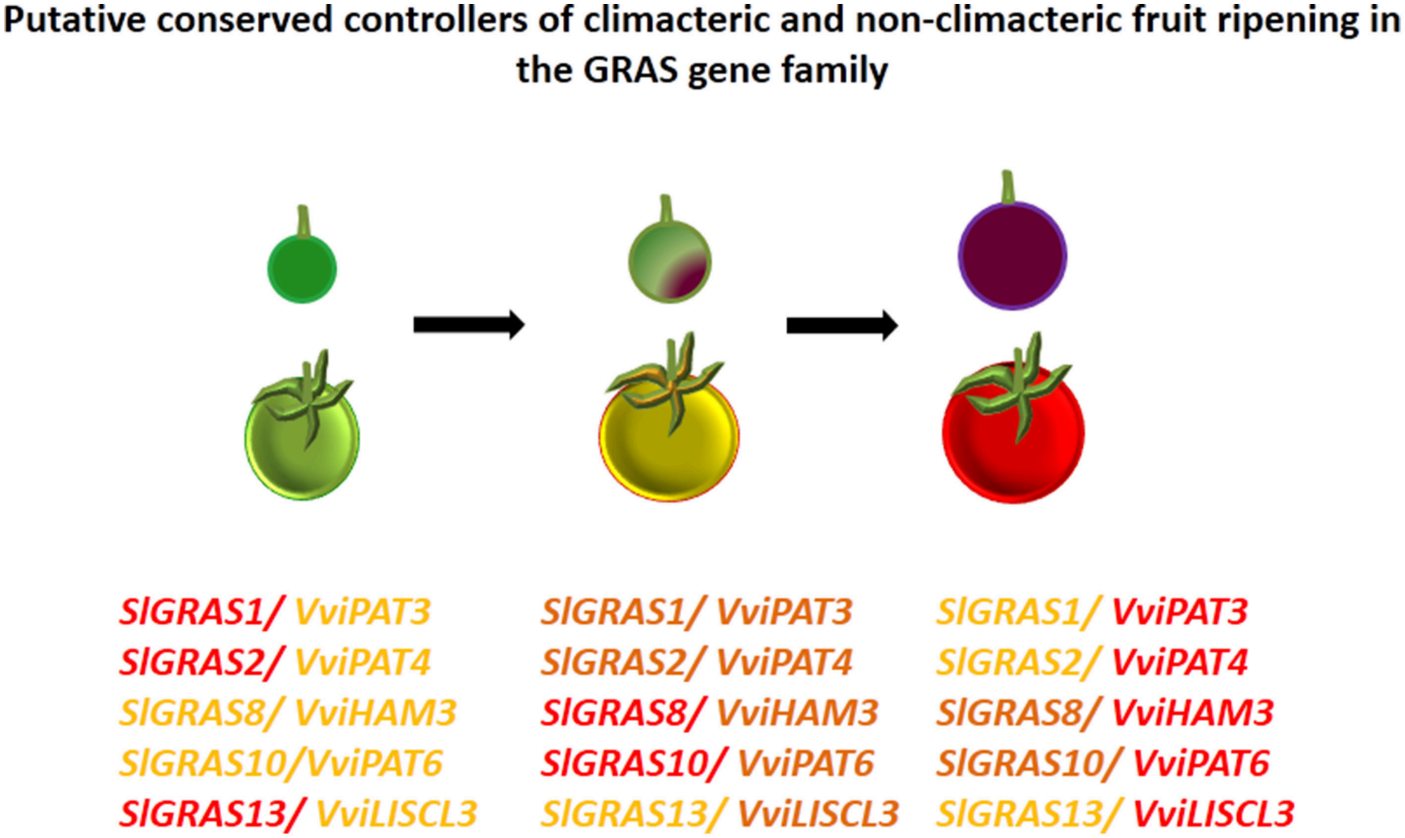
**Putative conserved controllers of climacteric and non-climacteric fruit ripening in the GRAS gene family**. GRAS gene expression is reported for three ripening stages in grape and tomato (green, *véraison*/ breaker, and ripe). Light orange corresponds to lower levels of expression, dark orange to medium levels and red to higher levels. The data is presented considering the tendency of the orthologs across cultivars in grape and tomato.

Altogether, these observations could suggest the relevance of *GRAS* genes as regulators of the different stages of grape berry development. GRAS transcription factors have been previously associated with the control of tomato fruit ripening (Fujisawa et al., 2012). Authors suggested that *SlGRAS38* gene could play a role in fruit ripening due to its ripening-specific expression and direct transcriptional regulation by RIN. In tomato, a typical climacteric fruit, the MADS-box transcription factor RIN is one of the earliest-acting ripening regulators, required for both ethylene-dependent and ethylene- independent pathways. By contrast, *VviSH4*, the grapevine ortholog of *SlGRAS38*, did not seem to be involved in grapevine ripening. Since grape is a non-climacteric fruit in which ethylene does not play a central role in the regulation of ripening (reviewed by Fortes et al., 2015), a different transcriptional regulatory pathway of ripening could be expected. Still, common aspects between ripening pathways in both type of fruits can be observed. Grapevine *VviPAT3, VviPAT4*, and *VviPAT6* have expression patterns consistent with their involvement in berry ripening and their tomato orthologs, *SlGRAS1, SlGRAS2*, and *SlGRAS10* (respectively) were differentially expressed from mature green stage fruits to breaker stage fruits (Huang et al., 2015). The same holds true for *VviHAM3* and its tomato ortholog *SlGRAS8* as well as *VviLISCL3* and its ortholog *SlGRAS13* (Huang et al., 2015). Therefore, these grapevine *GRAS* genes (Figure 9) could likely be conserved and represent pivotal transcriptional regulators of fruit ripening in both climacteric and non-climacteric species.

#### Grapevine GRAS genes are putatively involved in stress and defense responses

Several GRAS proteins have been associated with a role in stress signaling (reviewed by Bolle, 2016). Arabidopsis *scr* and *shr* loss of function mutants were found to be hypersensitive to abscisic acid (ABA) and to high levels of glucose but were not affected by high salinity or osmotic stress (Cui, 2012). In grapevine *VviSHR1* expression seemed to be affected by ABA but not by salt (Figure 7). Interestingly, expression of *VviSHR1* decreased during grape ripening when glucose levels significantly increased. Moreover, *VviSHR1* may be involved in grapevine response against virus whereas *VviSCR1* was up-regulated in green berries upon Botrytis infection. In fact, GRAS genes seem to be expressed upon abiotic and biotic factors (reviewed by Bolle, 2016). Furthermore, *VviSHR1* co-expressed with genes involved in stress response (glutaredoxin family protein, subtilase). A poplar *GRAS* gene showing the highest identity to Arabidopsis *SCL7*, conferred salt and drought tolerance to this plant (Ma et al., 2010). The duplicated gene of *AtSCL7, AtSCL4*, is orthologous of the grapevine *VviLAS2* which was down-regulated in response to salt but up-regulated upon UV light and long day exposure. *VviLAS2* expression also decreased upon Botrytis and co-expressed with up to 11 genes possibly involved in biotic stress response (epoxide hydrolase 2, DEFENSE NO death 1). *VviLAS2* might be a negative regulator of expression of these genes.

Other grapevine *GRAS* genes were found to show differential stress responses. *VviRGA5* was recently shown to be up-regulated in grape berries at initial stage of fungal infection (Agudelo-Romero et al., 2015) and *VviRGA3* was down-regulated under abiotic stresses such as salt, water stress, ABA exposure, and high light. Inhibition of growth by DELLA subfamily genes has been proposed as a response to environmental variability (Harberd et al., 2009) so these transcription factors may play an important role in the regulation of abiotic and biotic stress response pathways by regulating growth. Furthermore, DELLA proteins control plant immune responses by modulating the balance of jasmonic acid and salicylic acid signaling (Navarro et al., 2008; Wild et al., 2012), growth regulators which involvement in stress responses is well-known.

The Arabidopsis GRAS protein SCL14 was shown to be essential for the activation of stress-inducible promoters (Fode et al., 2008). The closest grapevine homologs are V*viLISCL12* and *VviLISCL3* that were also up-regulated after biotic stress. *VviLISCL12* was recently shown to be up-regulated upon guazatine treatment, an inhibitor of polyamine catabolism (Agudelo-Romero et al., 2014). In rice, *OsGRAS2*, the ortholog of *AtSCL14* is involved in the regulation of drought stress response (Xu et al., 2015). Other grapevine *LISCL* genes could likely be involved in abiotic stress response namely *VviLISCL1* which was over-expressed after long exposure to water deficit and salt stress (Figure 7).

The *Brassica oleracea* gene *BoGRAS*, was up-regulated under heat stress (Park et al., 2013) and its grapevine ortholog, *VviPAT3*, was also over-expressed during biotic stress. The ortholog of *VviPAT3* in tomato*, SlGRAS1*, was also referred to be involved in biotic stress response (Mayrose et al., 2006). Moreover, *VviPAT4* might be a good candidate in regulating abiotic and biotic stress responses in grapevine since it was up-regulated under both conditions. In tomato *SlGRAS2*, the *VviPAT4* ortholog, was involved in hormone signaling and abiotic stress response (Huang et al., 2015). *VviHAM3* was also up-regulated during ripening, upon *Bois Noir* attacks, and in response to drought in the seed and shoot tip. Therefore, *VviHAM3* exhibited expression patterns that indicate a role in broad stress responses.

Altogether, the expression of several grapevine *GRAS* genes in response to several stress treatments highlights the wide involvement of this gene family in environmental adaptation, showing diverse responses under different environmental conditions and treatments (Huang et al., 2015). The same results were observed in tomato for the expression of many *SlGRAS* genes.

## Conclusions

GRAS transcription factors have been characterized in several species and were proven to be involved in diverse developmental processes and stress responses. However, their involvement in fruit ripening is only now starting to be disclosed. Grape berry development and ripening could be under control of *GRAS* genes, since the expression of many of them is modulated during this process. The involvement of grapevine *GRAS* genes in stress responses was also confirmed in this study. Both ripening and stress responses involved genes from new GRAS subfamilies identified in grapevine (GRASV1, GRASV2, GRASV3, SCL26, and GRAS8). Robust candidates for further functional analysis were established and compared with the results of a similar analysis recently performed in tomato, another fleshy fruit. Altogether this data may contribute to the improvement of fruit quality and resilience to biotic and abiotic stresses.

## Author contributions

AF and JG designed the study. JG, PA, RT, and AF analyzed the data. AF wrote the manuscript with valuable input from JG and JM. All the authors revised and approved the manuscript.

### Conflict of interest statement

The authors declare that the research was conducted in the absence of any commercial or financial relationships that could be construed as a potential conflict of interest.

## Supplementary material

Supplementary Image 1 **Structure and subfamily-specific motifs of GRAS proteins**. The size varies within the subfamily. Several proteins such as VviLISCL2 and VviLISCL7 present shorter N-terminal sequences. The protein VviSCL3b lacks the motifs PFYRE and SAW.

Supplementary Image 2 **Molecular phylogenetic analysis by Maximum Likelihood method between Grapevine and 15 plant species**. Lineage-specific groups can be noticed for Populus and Eucalyptus whereas Arabidopsis putatively lacks specific subgroups.

Supplementary Table 1 **Complete annotation of the grapevine GRAS genes**. Alternative Names correspond to previous annotation (8X and 12Xv0). The probesets ID for microarray platform are given for Genechips, Grapegen and Nimblegen. The Nimblegen ID is also the 12Xv1 ID.IEP: evidence code inferred by expression pattern. Positions are given for both the 12X v1 and v2 genome.

Supplementary Table 2 **List of genes co-expressed with GRAS genes**. GRAS genes are highlighted in yellow.

Supplementary Table 3 **GRAS genes expression in experiments related to ripening, abiotic stress and biotic stress**.

## References

[B1] Agudelo-Romero P. Ali K. Choi Y. H. Sousa L. Verpoorte R. Tiburcio A. F.. (2014). Perturbation of polyamine catabolism affects grape ripening of *Vitis vinifera* cv. Trincadeira. Plant Physiol. Biochem. 74, 141–155. 10.1016/j.plaphy.2013.11.002 24296250

[B2] Agudelo-Romero P. Erban A. Rego C. Carbonell-Bejerano P. Nascimento T. Sousa L.. (2015). Transcriptome and metabolome reprogramming in *Vitis vinifera* cv. Trincadeira berries upon infection with Botrytis cinerea. J. Exp. Bot. 66, 1769–1785. 10.1093/jxb/eru517 25675955 PMC4669548

[B3] Agudelo-Romero P. Erban A. Sousa L. Pais M. S. Kopka J. Fortes A. M. (2013). Search for transcriptional and metabolic markers of grape pre-ripening and ripening and insights into specific aroma development in three Portuguese cultivars. PLoS ONE 8:e60422. 10.1371/journal.pone.0060422 23565246 PMC3614522

[B4] Albertazzi G. Milc J. Caffagni A. Francia E. Roncaglia E. Ferrari F. (2009). Gene expression in grapevine cultivars in response to Bois Noir phytoplasma infection. Plant Sci. 176, 792–804. 10.1016/j.plantsci.2009.03.001

[B5] Bolle C. (2004). The role of GRAS proteins in plant signal transduction and development. Planta 218, 683–692. 10.1007/s00425-004-1203-z 14760535

[B6] Bolle C. (2016). Functional aspects of GRAS family proteins, in Plant Transcription Factors, Evolutionary, Structural, and Functional Aspects, ed Gonzalez D. H. (Cambridge: Elsevier), 295–311.

[B7] Bolle C. Koncz C. Chua N. H. (2000). PAT1, a new member of the GRAS family, is involved in phytochrome A signal transduction. Genes Dev. 14, 1269–1278. 10.1101/gad.14.10.1269 10817761 PMC316623

[B8] Carbonell-Bejerano P. Santa Maria E. Torres-Perez R. Royo C. Lijavetzky D. Bravo G.. (2013). Thermotolerance responses in ripening berries of *Vitis vinifera L. cv Muscat Hamburg*. Plant Cell Physiol. 54, 1200–1216. 10.1093/pcp/pct071 23659918

[B9] Carvalho L. C. Vilela B. J. Mullineaux P. M. Amancio S. (2011). Comparative transcriptomic profiling of *Vitis vinifera* under high light using a custom-made array and the Affymetrix GeneChip. Mol. Plant 4, 1038–1051. 10.1093/mp/ssr027 21498622

[B10] Chen K. Li H. Chen Y. Zheng Q. Li B. Li Z. (2015). TaSCL14, a novel wheat (Triticum aestivum L.) GRAS gene, regulates plant growth, photosynthesis, tolerance to photooxidative stress, and senescence. J. Genet. Genomics 42, 21–32. 10.1016/j.jgg.2014.11.002 25619599

[B11] Cramer G. R. Ergul A. Grimplet J. Tillett R. L. Tattersall E. A. Bohlman M. C.. (2007). Water and salinity stress in grapevines: early and late changes in transcript and metabolite profiles. Funct. Integr. Genomics 7, 111–134. 10.1007/s10142-006-0039-y 17136344

[B12] Cui H. (2012). Killing two birds with one stone: transcriptional regulators coordinate development and stress responses in plants. Plant Signal. Behav. 7, 701–703. 10.4161/psb.20283 22580500 PMC3442873

[B13] Cui H. Kong D. Liu X. Hao Y. (2014). SCARECROW, SCR-LIKE 23 and SHORT-ROOT control bundle sheath cell fate and function in *Arabidopsis thaliana*. Plant J. 78, 319–327. 10.1111/tpj.12470 24517883

[B14] Cui H. Levesque M. P. Vernoux T. Jung J. W. Paquette A. J. Gallagher K. L.. (2007). An evolutionarily conserved mechanism delimiting SHR movement defines a single layer of endodermis in plants. Science 316, 421–425. 10.1126/science.1139531 17446396

[B15] Day R. B. Tanabe S. Koshioka M. Mitsui T. Itoh H. Ueguchi-Tanaka M.. (2004). Two rice GRAS family genes responsive to N -acetylchitooligosaccharide elicitor are induced by phytoactive gibberellins: evidence for cross-talk between elicitor and gibberellin signaling in rice cells. Plant Mol. Biol. 54, 261–272. 10.1023/B:PLAN.0000028792.72343.ee 15159627

[B16] Deluc L. G. Grimplet J. Wheatley M. D. Tillett R. L. Quilici D. R. Osborne C.. (2007). Transcriptomic and metabolite analyses of Cabernet Sauvignon grape berry development. BMC Genomics 8:429. 10.1186/1471-2164-8-429 18034876 PMC2220006

[B17] Diaz-Riquelme J. Grimplet J. Martinez-Zapater J. M. Carmona M. J. (2012). Transcriptome variation along bud development in grapevine (*Vitis vinifera* L.). BMC Plant Biol. 12:181. 10.1186/1471-2229-12-181 23035802 PMC3519583

[B18] Edgar R. C. (2004). MUSCLE: multiple sequence alignment with high accuracy and high throughput. Nucleic Acids Res. 32, 1792–1797. 10.1093/nar/gkh340 15034147 PMC390337

[B19] Espinoza C. Vega A. Medina C. Schlauch K. Cramer G. Arce-Johnson P. (2007). Gene expression associated with compatible viral diseases in grapevine cultivars. Funct. Integr. Genomics 7, 95–110. 10.1007/s10142-006-0031-6 16775684

[B20] Fasoli M. Dal Santo S. Zenoni S. Tornielli G. B. Farina L. Zamboni A.. (2012). The grapevine expression atlas reveals a deep transcriptome shift driving the entire plant into a maturation program. Plant Cell 24, 3489–3505. 10.1105/tpc.112.100230 22948079 PMC3480284

[B21] Felsenstein J. (1985). Confidence limits on phylogenies: an approach using the bootstrap. Evolution 39, 783–791. 10.2307/2408678 28561359

[B22] Fode B. Siemsen T. Thurow C. Weigel R. Gatz C. (2008). The Arabidopsis GRAS protein SCL14 interacts with class II TGA transcription factors and is essential for the activation of stress-inducible promoters. Plant Cell 20, 3122–3135. 10.1105/tpc.108.058974 18984675 PMC2613660

[B23] Fortes A. M. Agudelo-Romero P. Silva M. S. Ali K. Sousa L. Maltese F.. (2011). Transcript and metabolite analysis in Trincadeira cultivar reveals novel information regarding the dynamics of grape ripening. BMC Plant Biol. 11:149. 10.1186/1471-2229-11-149 22047180 PMC3215662

[B24] Fortes A. M. Teixeira R. T. Agudelo-Romero P. (2015). Complex interplay of hormonal signals during grape berry ripening. Molecules 20, 9326–9343. 10.3390/molecules20059326 26007186 PMC6272489

[B25] Fu X. Richards D. E. Ait-Ali T. Hynes L. W. Ougham H. Peng J.. (2002). Gibberellin-mediated proteasome-dependent degradation of the barley DELLA protein SLN1 repressor. Plant Cell 14, 3191–3200. 10.1105/tpc.006197 12468736 PMC151211

[B26] Fujisawa M. Shima Y. Higuchi N. Nakano T. Koyama Y. Kasumi T.. (2012). Direct targets of the tomato-ripening regulator RIN identified by transcriptome and chromatin immunoprecipitation analyses. Planta 235, 1107–1122. 10.1007/s00425-011-1561-2 22160566

[B27] Fung R. W. Gonzalo M. Fekete C. Kovacs L. G. He Y. Marsh E.. (2008). Powdery mildew induces defense-oriented reprogramming of the transcriptome in a susceptible but not in a resistant grapevine. Plant Physiol. 146, 236–249. 10.1104/pp.107.108712 17993546 PMC2230561

[B28] Gao M. J. Parkin I. Lydiate D. Hannoufa A. (2004). An auxin-responsive SCARECROW-like transcriptional activator interacts with histone deacetylase. Plant Mol. Biol. 55, 417–431. 10.1007/s11103-004-0892-9 15604690

[B29] Greb T. Clarenz O. Schafer E. Muller D. Herrero R. Schmitz G.. (2003). Molecular analysis of the LATERAL SUPPRESSOR gene in Arabidopsis reveals a conserved control mechanism for axillary meristem formation. Genes Dev. 17, 1175–1187. 10.1101/gad.260703 12730136 PMC196050

[B30] Grimplet J. Adam-Blondon A.-F. Bert P.-F. Bitz O. Cantu D. Davies C.. (2014). The grapevine gene nomenclature system. BMC Genomics 15:1077. 10.1186/1471-2164-15-1077 25481684 PMC4299395

[B31] Grimplet J. Deluc L. G. Tillett R. L. Wheatley M. D. Schlauch K. A. Cramer G. R.. (2007). Tissue-specific mRNA expression profiling in grape berry tissues. BMC Genomics 8:187. 10.1186/1471-2164-8-187 17584945 PMC1925093

[B32] Grimplet J. Van Hemert J. Carbonell-Bejerano P. Diaz-Riquelme J. Dickerson J. Fennell A.. (2012). Comparative analysis of grapevine whole-genome gene predictions, functional annotation, categorization and integration of the predicted gene sequences. BMC Res. Notes 5:213. 10.1186/1756-0500-5-213 22554261 PMC3419625

[B33] Harberd N. P. Belfield E. Yasumura Y. (2009). The angiosperm gibberellin-GID1-DELLA growth regulatory mechanism: how an “inhibitor of an inhibitor” enables flexible response to fluctuating environments. Plant Cell 21, 1328–1339. 10.1105/tpc.109.066969 19470587 PMC2700538

[B34] Heckmann A. B. Lombardo F. Miwa H. Perry J. A. Bunnewell S. Parniske M.. (2006). *Lotus japonicus* nodulation requires two GRAS domain regulators, one of which is functionally conserved in a non-legume. Plant Physiol. 142, 1739–1750. 10.1104/pp.106.089508 17071642 PMC1676053

[B35] Helariutta Y. Fukaki H. Wysocka-Diller J. Nakajima K. Jung J. Sena G.. (2000). The SHORT-ROOT gene controls radial patterning of the Arabidopsis root through radial signaling. Cell 101, 555–567. 10.1016/S0092-8674(00)80865-X 10850497

[B36] Hirsch S. Oldroyd G. E. (2009). GRAS-domain transcription factors that regulate plant development. Plant Signal. Behav. 4, 698–700. 10.4161/psb.4.8.9176 19820314 PMC2801379

[B37] Huang W. Xian Z. Kang X. Tang N. Li Z. (2015). Genome-wide identification, phylogeny and expression analysis of GRAS gene family in tomato. BMC Plant Biol. 15:209. 10.1186/s12870-015-0590-6 26302743 PMC4549011

[B38] Ikeda A. Ueguchi-Tanaka M. Sonoda Y. Kitano H. Koshioka M. Futsuhara Y.. (2001). slender rice, a constitutive gibberellin response mutant, is caused by a null mutation of the SLR1 gene, an ortholog of the height-regulating gene GAI/RGA/RHT/D8. Plant Cell 13, 999–1010. 10.1105/tpc.13.5.999 11340177 PMC135552

[B39] Itoh H. Ueguchi-Tanaka M. Sato Y. Ashikari M. Matsuoka M. (2002). The gibberellin signaling pathway is regulated by the appearance and disappearance of SLENDER RICE1 in nuclei. Plant Cell 14, 57–70. 10.1105/tpc.010319 11826299 PMC150551

[B40] Jaillon O. Aury J. M. Noel B. Policriti A. Clepet C. Casagrande A.. (2007). The grapevine genome sequence suggests ancestral hexaploidization in major angiosperm phyla. Nature 449, 463–467. 10.1038/nature06148 17721507

[B41] Jones D. T. Taylor W. R. Thornton J. M. (1992). The rapid generation of mutation data matrices from protein sequences. Bioinformatics 8, 275–282. 10.1093/bioinformatics/8.3.275 1633570

[B42] Kalo P. Gleason C. Edwards A. Marsh J. Mitra R. M. Hirsch S.. (2005). Nodulation signaling in legumes requires NSP2, a member of the GRAS family of transcriptional regulators. Science 308, 1786–1789. 10.1126/science.1110951 15961668

[B43] Karlova R. van Haarst J. C. Maliepaard C. van de Geest H. Bovy A. G. Lammers M.. (2013). Identification of microRNA targets in tomato fruit development using high-throughput sequencing and degradome analysis. J. Exp. Bot. 64, 1863–1878. 10.1093/jxb/ert049 23487304 PMC3638818

[B44] Lee M. H. Kim B. Song S. K. Heo J. O. Yu N. I. Lee S. A.. (2008). Large-scale analysis of the GRAS gene family in *Arabidopsis thaliana*. Plant Mol. Biol. 67, 659–670. 10.1007/s11103-008-9345-1 18500650

[B45] Li X. Qian Q. Fu Z. Wang Y. Xiong G. Zeng D.. (2003). Control of tillering in rice. Nature 422, 618–621. 10.1038/nature01518 12687001

[B46] Licausi F. Giorgi F. M. Zenoni S. Osti F. Pezzotti M. Perata P. (2010). Genomic and transcriptomic analysis of the AP2/ERF superfamily in *Vitis vinifera*. BMC Genomics 11:719. 10.1186/1471-2164-11-719 21171999 PMC3022922

[B47] Lijavetzky D. Carbonell-Bejerano P. Grimplet J. Bravo G. Flores P. Fenoll J.. (2012). Berry flesh and skin ripening features in *Vitis vinifera* as assessed by transcriptional profiling. PLoS ONE 7:e39547. 10.1371/annotation/fd93800a-3b3c-484d-97a9-190043309e4b 22768087 PMC3386993

[B48] Liu X. Widmer A. (2014). Genome-wide comparative analysis of the GRAS gene family in populus, Arabidopsis and rice. Plant Mol. Biol. Rep. 32, 1129–1145. 10.1007/s11105-014-0721-5

[B49] Lu J. Wang T. Xu Z. Sun L. Zhang Q. (2015). Genome-wide analysis of the GRAS gene family in *Prunus mume*. Mol. Genet. Genomics 290, 303–317. 10.1007/s00438-014-0918-1 25245166

[B50] Lund S. T. Peng F. Y. Nayar T. Reid K. E. Schlosser J. (2008). Gene expression analyses in individual grape (*Vitis vinifera* L.) berries during ripening initiation reveal that pigmentation intensity is a valid indicator of developmental staging within the cluster. Plant Mol. Biol. 68, 301–315. 10.1007/s11103-008-9371-z 18642093

[B51] Ma H. S. Liang D. Shuai P. Xia X. L. Yin W. L. (2010). The salt- and drought-inducible poplar GRAS protein SCL7 confers salt and drought tolerance in *Arabidopsis thaliana*. J. Exp. Bot. 61, 4011–4019. 10.1093/jxb/erq217 20616154 PMC2935874

[B52] Mayrose M. Ekengren S. K. Melech-Bonfil S. Martin G. B. Sessa G. (2006). A novel link between tomato GRAS genes, plant disease resistance and mechanical stress response. Mol. Plant Pathol. 7, 593–604. 10.1111/j.1364-3703.2006.00364.x 20507472

[B53] Morohashi K. Minami M. Takase H. Hotta Y. Hiratsuka K. (2003). Isolation and characterization of a novel GRAS gene that regulates meiosis-associated gene expression. J. Biol. Chem. 278, 20865–20873. 10.1074/jbc.M301712200 12657631

[B54] Moxon S. Jing R. Szittya G. Schwach F. Rusholme Pilcher R. L. Moulton V.. (2008). Deep sequencing of tomato short RNAs identifies microRNAs targeting genes involved in fruit ripening. Genome Res. 18, 1602–1609. 10.1101/gr.080127.108 18653800 PMC2556272

[B55] Navarro L. Bari R. Achard P. Lison P. Nemri A. Harberd N. P.. (2008). DELLAs control plant immune responses by modulating the balance of jasmonic acid and salicylic acid signaling. Curr. Biol. 18, 650–655. 10.1016/j.cub.2008.03.060 18450451

[B56] Obayashi T. Kinoshita K. (2009). Rank of correlation coefficient as a comparable measure for biological significance of gene coexpression. DNA Res. 16, 249–260. 10.1093/dnares/dsp016 19767600 PMC2762411

[B57] Okonechnikov K. Golosova O. Fursov M. team U. (2012). Unipro UGENE: a unified bioinformatics toolkit. Bioinformatics 28, 1166–1167. 10.1093/bioinformatics/bts091 22368248

[B58] Oldroyd G. E. (2013). Speak, friend, and enter: signalling systems that promote beneficial symbiotic associations in plants. Nat. Rev. Microbiol. 11, 252–263. 10.1038/nrmicro2990 23493145

[B59] Park H. J. Jung W. Y. Lee S. S. Song J. H. Kwon S. Y. Kim H.. (2013). Use of heat stress responsive gene expression levels for early selection of heat tolerant cabbage (*Brassica oleracea* L.). Int. J. Mol. Sci. 14, 11871–11894. 10.3390/ijms140611871 23736694 PMC3709761

[B60] Peng J. Carol P. Richards D. E. King K. E. Cowling R. J. Murphy G. P.. (1997). The Arabidopsis GAI gene defines a signaling pathway that negatively regulates gibberellin responses. Genes Dev. 11, 3194–3205. 10.1101/gad.11.23.3194 9389651 PMC316750

[B61] Pilati S. Perazzolli M. Malossini A. Cestaro A. Dematte L. Fontana P.. (2007). Genome-wide transcriptional analysis of grapevine berry ripening reveals a set of genes similarly modulated during three seasons and the occurrence of an oxidative burst at veraison. BMC Genomics 8:428. 10.1186/1471-2164-8-428 18034875 PMC2228314

[B62] Pontin M. A. Piccoli P. N. Francisco R. Bottini R. Martinez-Zapater J. M. Lijavetzky D. (2010). Transcriptome changes in grapevine (*Vitis vinifera L*.) cv. Malbec leaves induced by ultraviolet-B radiation. BMC Plant Biol 10:224. 10.1186/1471-2229-10-224 20959019 PMC3017828

[B63] Prince V. E. Pickett F. B. (2002). Splitting pairs: the diverging fates of duplicated genes. Nat. Rev. Genet. 3, 827–837. 10.1038/nrg928 12415313

[B64] Pysh L. D. Wysocka-Diller J. W. Camilleri C. Bouchez D. Benfey P. N. (1999). The GRAS gene family in Arabidopsis: sequence characterization and basic expression analysis of the SCARECROW-LIKE genes. Plant J. 18, 111–119. 10.1046/j.1365-313X.1999.00431.x 10341448

[B65] Royo C. Carbonell-Bejerano P. Torres-Perez R. Nebish A. Martinez O. Rey M.. (2016). *Developmental*, transcriptome, and genetic alterations associated with parthenocarpy in the grapevine seedless somatic variant Corinto bianco. J. Exp. Bot. 67, 259–273. 10.1093/jxb/erv452 26454283

[B66] Schumacher K. Schmitt T. Rossberg M. Schmitz G. Theres K. (1999). The Lateral suppressor (Ls) gene of tomato encodes a new member of the VHIID protein family. Proc. Natl. Acad. Sci. U.S.A. 96, 290–295. 10.1073/pnas.96.1.290 9874811 PMC15132

[B67] Silverstone A. L. Ciampaglio C. N. Sun T. (1998). The Arabidopsis RGA gene encodes a transcriptional regulator repressing the gibberellin signal transduction pathway. Plant Cell 10, 155–169. 10.1105/tpc.10.2.155 9490740 PMC143987

[B68] Song X. M. Liu T. K. Duan W. K. Ma Q. H. Ren J. Wang Z.. (2014). Genome-wide analysis of the GRAS gene family in Chinese cabbage (*Brassica rapa* ssp. pekinensis). Genomics 103, 135–146. 10.1016/j.ygeno.2013.12.004 24365788

[B69] Sreekantan L. Mathiason K. Grimplet J. Schlauch K. Dickerson J. A. Fennell A. Y. (2010). Differential floral development and gene expression in grapevines during long and short photoperiods suggests a role for floral genes in dormancy transitioning. Plant Mol. Biol. 73, 191–205. 10.1007/s11103-010-9611-x 20151315

[B70] Sterck L. Billiau K. Abeel T. Rouze P. Van de Peer Y. (2012). ORCAE: online resource for community annotation of eukaryotes. Nat. Methods 9:1041. 10.1038/nmeth.2242 23132114

[B71] Stuurman J. Jaggi F. Kuhlemeier C. (2002). Shoot meristem maintenance is controlled by a GRAS-gene mediated signal from differentiating cells. Genes Dev. 16, 2213–2218. 10.1101/gad.230702 12208843 PMC186665

[B72] Tamura K. Stecher G. Peterson D. Filipski A. Kumar S. (2013). MEGA6: molecular evolutionary genetics analysis version 6.0. Mol. Biol. Evol. 30, 2725–2729. 10.1093/molbev/mst197 24132122 PMC3840312

[B73] Tattersall E. A. Grimplet J. DeLuc L. Wheatley M. D. Vincent D. Osborne C.. (2007). Transcript abundance profiles reveal larger and more complex responses of grapevine to chilling compared to osmotic and salinity stress. Funct. Integr. Genomics 7, 317–333. 10.1007/s10142-007-0051-x 17578611

[B74] Tian C. Wan P. Sun S. Li J. Chen M. (2004). Genome-wide analysis of the GRAS gene family in rice and Arabidopsis. Plant Mol. Biol. 54, 519–532. 10.1023/B:PLAN.0000038256.89809.57 15316287

[B75] Tillett R. L. Ergul A. Albion R. L. Schlauch K. A. Cramer G. R. Cushman J. C. (2011). Identification of tissue-specific, abiotic stress-responsive gene expression patterns in wine grape (*Vitis vinifera L*.) based on curation and mining of large-scale EST data sets. BMC Plant Biol. 11:86. 10.1186/1471-2229-11-86 21592389 PMC3224124

[B76] Tong H. Jin Y. Liu W. Li F. Fang J. Yin Y.. (2009). DWARF AND LOW-TILLERING, a new member of the GRAS family, plays positive roles in brassinosteroid signaling in rice. Plant J. 58, 803–816. 10.1111/j.1365-313X.2009.03825.x 19220793

[B77] Torres-Galea P. Hirtreiter B. Bolle C. (2013). Two GRAS proteins, SCARECROW-LIKE21 and PHYTOCHROME A SIGNAL TRANSDUCTION1, function cooperatively in phytochrome A signal transduction. Plant Physiol. 161, 291–304. 10.1104/pp.112.206607 23109688 PMC3532260

[B78] Torres-Galea P. Huang L. F. Chua N. H. Bolle C. (2006). The GRAS protein SCL13 is a positive regulator of phytochrome-dependent red light signaling, but can also modulate phytochrome A responses. Mol. Genet. Genomics 276, 13–30. 10.1007/s00438-006-0123-y 16680434

[B79] Vega A. Gutierrez R. A. Pena-Neira A. Cramer G. R. Arce-Johnson P. (2011). Compatible GLRaV-3 viral infections affect berry ripening decreasing sugar accumulation and anthocyanin biosynthesis in *Vitis vinifera*. Plant Mol. Biol. 77, 261–274. 10.1007/s11103-011-9807-8 21786204

[B80] Velasco R. Zharkikh A. Troggio M. Cartwright D. A. Cestaro A. Pruss D.. (2007). A high quality draft consensus sequence of the genome of a heterozygous grapevine variety. PLoS ONE 2:e1326. 10.1371/journal.pone.0001326 18094749 PMC2147077

[B81] Wang L. Mai Y. X. Zhang Y. C. Luo Q. Yang H. Q. (2010). MicroRNA171c-targeted SCL6-II, SCL6-III, and SCL6-IV genes regulate shoot branching in Arabidopsis. Mol. Plant 3, 794–806. 10.1093/mp/ssq042 20720155

[B82] Wild M. Daviere J. M. Cheminant S. Regnault T. Baumberger N. Heintz D.. (2012). The Arabidopsis DELLA RGA-LIKE3 is a direct target of MYC2 and modulates jasmonate signaling responses. Plant Cell 24, 3307–3319. 10.1105/tpc.112.101428 22892320 PMC3462633

[B83] Wu N. Zhu Y. Song W. Li Y. Yan Y. Hu Y. (2014). Unusual tandem expansion and positive selection in subgroups of the plant GRAS transcription factor superfamily. BMC Plant Biol. 14:373. 10.1186/s12870-014-0373-5 25524588 PMC4279901

[B84] Xu K. Chen S. Li T. Ma X. Liang X. Ding X.. (2015). OsGRAS23, a rice GRAS transcription factor gene, is involved in drought stress response through regulating expression of stress-responsive genes. BMC Plant Biol. 15:141. 10.1186/s12870-015-0532-3 26067440 PMC4465154

[B85] Xue L. Cui H. Buer B. Vijayakumar V. Delaux P. M. Junkermann S.. (2015). Network of GRAS transcription factors involved in the control of arbuscule development in *Lotus japonicus*. Plant Physiol. 167, 854–871. 10.1104/pp.114.255430 25560877 PMC4348782

[B86] Zentella R. Zhang Z. L. Park M. Thomas S. G. Endo A. Murase K.. (2007). Global analysis of della direct targets in early gibberellin signaling in Arabidopsis. Plant Cell 19, 3037–3057. 10.1105/tpc.107.054999 17933900 PMC2174696

[B87] Zhang D. Iyer L. M. Aravind L. (2012). Bacterial GRAS domain proteins throw new light on gibberellic acid response mechanisms. Bioinformatics 28, 2407–2411. 10.1093/bioinformatics/bts464 22829623 PMC3463117

