# Supplementary figures and images for: Structural and Functional Analysis of the GRAS Gene Family in Grapevine Indicates a Role of GRAS Proteins in the Control of Development and Stress Responses
Source: Front Plant Sci. 2016 Mar 30;7:353. doi: 10.3389/fpls.2016.00353 (PMC4811876; doi:10.3389/fpls.2016.00353)

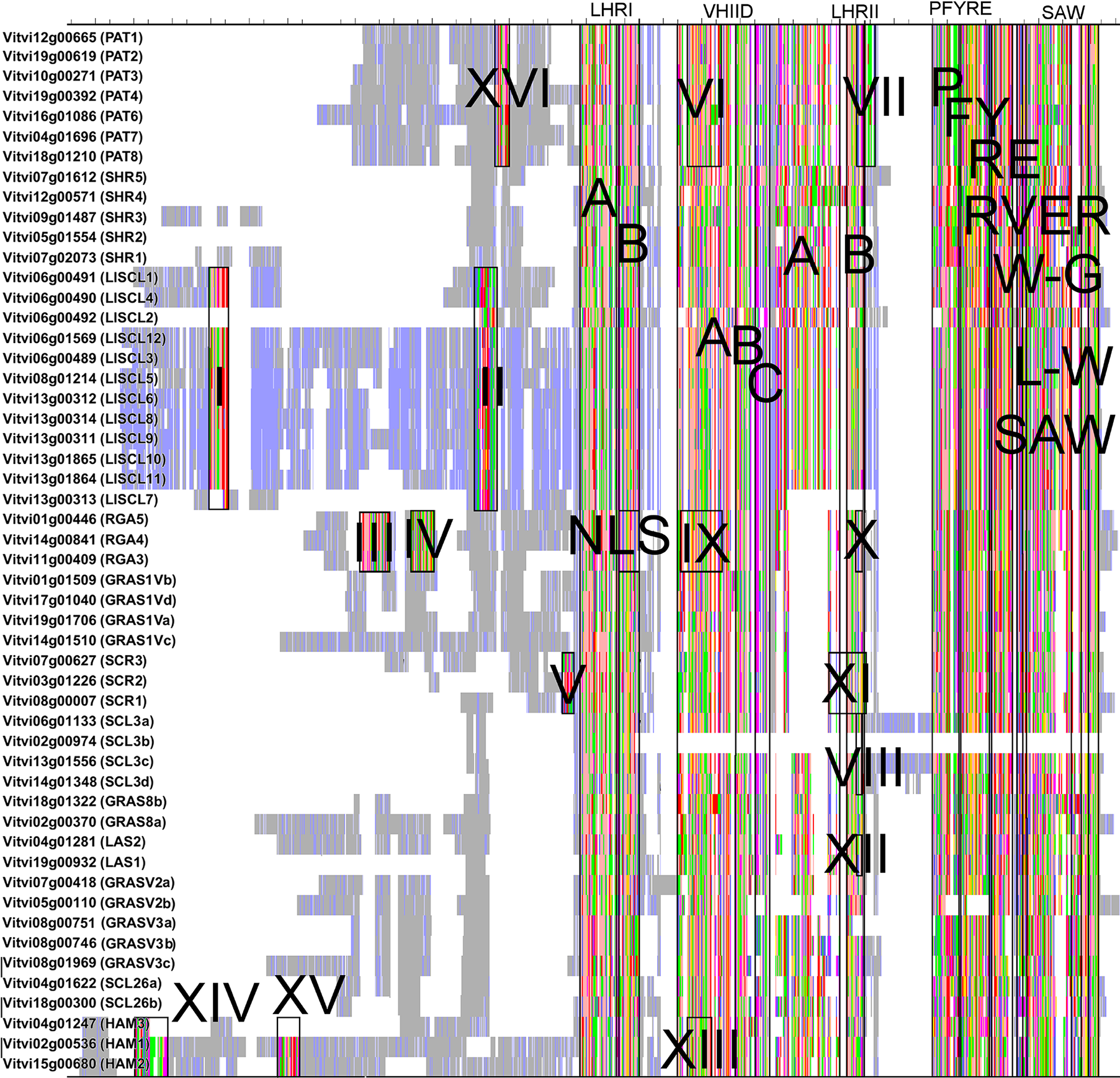

Supplement: Supplementary Image 1 — Structure and subfamily-specific motifs of GRAS proteins. The size varies within the subfamily. Several proteins such as VviLISCL2 and VviLISCL7 present shorter N-terminal sequences. The protein VviSCL3b lacks the motifs PFYRE and SAW. [file Image1.TIFF]
